# Comparative Genomics of Ten Solanaceous Plastomes

**DOI:** 10.1155/2014/424873

**Published:** 2014-11-17

**Authors:** Harpreet Kaur, Bhupinder Pal Singh, Harpreet Singh, Avinash Kaur Nagpal

**Affiliations:** ^1^Department of Botanical and Environmental Sciences, Guru Nanak Dev University, Amritsar 143005, India; ^2^Department of Bioinformatics, Hans Raj Mahila Maha Vidyalaya, Jalandhar 144008, India

## Abstract

Availability of complete plastid genomes of ten solanaceous species, *Atropa belladonna*, *Capsicum annuum*, *Datura stramonium*, *Nicotiana sylvestris*, *Nicotiana tabacum*, *Nicotiana tomentosiformis*, *Nicotiana undulata*, *Solanum bulbocastanum*, *Solanum lycopersicum*, and *Solanum tuberosum* provided us with an opportunity to conduct their *in silico* comparative analysis in depth. The size of complete chloroplast genomes and LSC and SSC regions of three species of *Solanum* is comparatively smaller than that of any other species studied till date (exception: SSC region of *A. belladonna*). AT content of coding regions was found to be less than noncoding regions. A duplicate copy of trnH gene in *C. annuum* and two alternative tRNA genes for proline in *D. stramonium* were observed for the first time in this analysis. Further, homology search revealed the presence of rps19 pseudogene and infA genes in *A. belladonna* and *D. stramonium*, a region identical to rps19 pseudogene in *C. annum* and orthologues of sprA gene in another six species. Among the eighteen intron-containing genes, 3 genes have two introns and 15 genes have one intron. The longest insertion was found in accD gene in *C. annuum*. Phylogenetic analysis using concatenated protein coding sequences gave two clades, one for *Nicotiana* species and another for *Solanum*, *Capsicum*, *Atropa*, and *Datura*.

## 1. Introduction

Chloroplasts are essential cellular organelles within plant cells possessing the enzymatic machinery for the process of photosynthesis which provides essential energy to plants. Besides photosynthesis, chloroplasts are also involved in biosynthesis of fatty acids, amino acids, pigments, and vitamins [1, 2]. Despite enormous divergence in whole plant form and habitat, chloroplast structure and function have remained remarkably conserved which might be due to intense evolutionary selection pressures associated with the functional requirements of photosynthesis [3–7]. The chloroplast genome is actually a reduced genome derived from a cyanobacterial ancestor that was captured early in the evolution of the eukaryotic cell [8, 9]. Among the three genomes of the plant cell, the plastome is the most gene dense with more than 100 genes in a genome of only 120 to 210 kb [10]. In the last two decades, the nucleotide sequences of large number of plastid genomes have been published leading to better understanding of their organization and evolution [2, 11, 12]. Currently, about 470 eukaryotic chloroplast genomes have been sequenced completely (http://www.ncbi.nlm.nih.gov/genomes/GenomesHome.cgi?taxid=2759&hopt=html) with the best representation from flowering plants.

Most land plant chloroplast genomes are composed of a single circular chromosome with a quadripartite structure which includes two copies of an inverted repeat (IR) region that separates the large and small single copy regions (LSC and SSC). Genes of chloroplast genomes of higher plants can be divided into three broad categories [13, 14]. In the first, there are genetic system genes encoding for rRNAs, tRNAs, ribosomal proteins, and RNA polymerase subunits. The second category is comprised of genes for photosynthesis which encode subunits of the two photosystems, the cytochrome b6f complex and the ATP synthase. Open reading frames (*orfs*) of unknown function constitute the third category. Besides, there are some other genes coding for different kinds of proteins including infA, matK, clpP, cemA, accD, and ccsA. Although overall chloroplast genome organization is highly conserved among taxa, structural rearrangements due to inversions have been reported in different taxa like Campanulaceae [15], Cyatheaceae [16], Fabaceae [17], Funariaceae [18], Geraniaceae [19], Onagraceae [20], and Poaceae [21, 22]. Besides structural rearrangements, sequence polymorphisms have also been reported in some cereals [23, 24] and* Oenothera* species [20]. These studies revealed that highly divergent sequences were concentrated in specific regions called “hotspots.” Such sequence polymorphisms have been used to derive phylogenetic relationships among species.

Solanaceae is an important family of dicots comprising more than 3000 species placed within about 90 genera. It is an ethnobotanical family and is extensively utilized by humans and has recently become a model of comparative and evolutionary genomics research. Few efforts have been made to study the variations in chloroplast genomes of Solanaceae family by using* in silico* tools. Most of these attempts have been concentrated on comparison of newly sequenced chloroplast genome with the available complete chloroplast genomes from some members of this family [25–29]. The availability of complete nucleotide sequences of plastid genomes of ten solanaceous species,* Atropa belladonna* (NC_004561.1; [30]),* Capsicum annuum* (NC_018552.1; [29]),* Datura stramonium* (NC_018117.1; Li et al. (unpublished)),* Nicotiana sylvestris* (NC_007500.1; [28]),* Nicotiana tabacum* (NC_001879.2; [31]),* Nicotiana tomentosiformis* (NC_007602.1; [28]),* Nicotiana undulata* (NC_016068.1; [32]),* Solanum bulbocastanum* (NC_007943.1; [26]),* Solanum lycopersicum* (NC_007898.2; [27]), and* Solanum tuberosum* (NC_008096.2; [33]), provided us with an opportunity to conduct their* in silico* comparative analysis in depth. Hence, the present study is an attempt to compare the genome organization, structure, and coding capacity of chloroplast genomes of ten solanaceous species. The study focuses on length mutations, intron-containing genes, grouping of genes in different identity classes based on pairwise comparison of individual genes, and InDel analysis of divergent genes.

## 2. Materials and Methods

### 2.1. Sequence Analysis

Whole chloroplast genome sequences as well as individual gene and protein sequences of ten solanaceous species were obtained from “Organelle Genome Resources” section of NCBI in Genbank as well as in Fasta format. Sequence regions corresponding to various genomic features including genes, exons, introns, and cds were specifically extracted from the Genbank files using Extractfeat, Extractseq, and Featcopy programs from Jemboss package. AT percentage for different genomic regions was calculated using Wordcount and Union programs from Jemboss package. Pairwise comparison of gene sequences was done by using NCBI BLAST program and multiple sequence alignment of nucleotide as well as protein sequences was done by using ClustalW. Alignments of protein sequences for some of the genes were manually edited in correspondence to InDels observed in alignments of their nucleotide sequences.

### 2.2. Phylogenetic Analysis of Concatenated Protein-Coding Genes

75 protein-coding genes of plastomes of ten solanaceous species and two outgroup species (*Daucus carota* and* Coffea arabica*) were selected for phylogenetic analysis from the total of 79 classified protein-coding genes excluding accD, rpl20, ycf1, and ycf15. Ycf15 was excluded due to its absence on the plastome of both outgroup species chosen while the other three were not included in the phylogenetic analysis due to their high levels of variation. Multiple sequence alignment of each gene was obtained using ClustalW (https://www.ebi.ac.uk/Tools/msa/clustalw2/). These alignments were then concatenated using standalone BIOEDIT version 7.25 (http://www.mbio.ncsu.edu/bioedit/bioedit.html) and maximum likelihood phylogenetic tree with 500 bootstrap iterations was constructed using PhyMLv3.0 (http://www.atgc-montpellier.fr/phyml/). A graphical view of tree was generated using Archaeopteryx 0.988 SR (https://sites.google.com/site/cmzmasek/home/software/archaeopteryx).

## 3. Results and Discussion

### 3.1. Comparison of Properties of Chloroplast Genomes

Comparison of the properties of plastid genomes of ten solanaceous species with respect to their genome size (size of complete plastid genome and LSC, SSC, and IR regions); percent coding regions, introns, and intergenic regions; AT content of overall plastid genomes as well as coding and noncoding regions is presented in Table 1. The total plastid genome size ranged from 155296 bp (*S. tuberosum*) to 156781 bp (*C. annuum*). The large size of plastome of* C. annuum* can be attributed to large LSC region as compared to other species. On the contrary, size of SSC region in* C. annuum* was the least as compared to other species. The largest size of IR region was in* A. belladonna*. Among four* Nicotiana* species studied,* N. sylvestris* and* N. tabacum* were almost identical to each other with respect to size of complete genome (difference of only 2 bps) or LSC, SSC, or IR regions compared with plastome of any other species studied. However the percent coding region was slightly more for* N. sylvestris* (61.49%) than in* N. tabacum* (61.12%). The size of complete chloroplast genome and LSC and SSC regions of three species of* Solanum* is comparatively smaller than that of any other species studied except for* A. belladonna* where size of SSC region was the smallest (18008 bp). However the size of IR region of* Solanum* species is larger as compared to* Nicotiana* species. Coding region percentage was found to be higher in* Nicotiana* species as compared to all other species with maximum for* N. undulata* (63.12%) and minimum for* S. tuberosum* (58.45%). Maximum of 12.8% of the plastome was shown to be introns for* S. bulbocastanum* whereas minimum intron percentage (11.62%) was observed for* D. stramonium*. Maximum percentage (29.19%) of intergenic region was observed in* D. stramonium* and minimum (24.19%) was observed in* N. undulata.* The AT content of noncoding regions was found to be higher as compared to coding regions for all the ten species studied. Similarly, protein-coding regions have shown higher content of AT base pairs as compared to RNA coding genes which can be explained by the requirement of more GC base pairs for proper folding of highly structured ribosomal RNAs and tRNAs [13–27]. Comparison of AT content in LSC, SSC, and IR regions reveals that AT content was the highest in SSC regions and the lowest in IR regions. Some earlier studies have also shown similar distribution of AT content in LSC, SSC, and IR regions with the lowest AT content in IR region and the highest AT content in SSC region [2, 27, 34, 35]. The low AT content of IR regions reflects low AT content in the four ribosomal RNA genes in this region.

### 3.2. Gene Content of Solanaceous Chloroplast Genomes

The genes present in different regions of the plastid genomes are highly conserved except for several open reading frames [6, 26, 36]. There are typically 111 genes, 5 hypothetical chloroplast reading frames (ycfs), and few open reading frames (orfs). Some of our unique findings have been discussed below.

- The trnP-GGG which codes for tRNA for proline was annotated only in* D. stramonium* whereas its alternative code trnP-UGG was annotated in all other species including* D. stramonium* (NC_018117.1; Li et al. (unpublished)). We mined all the species for similar sequence by BLAST search but no similar sequence was found in any other species. Gene trnH was only reported to be trnH coding gene in* C. annuum*. In all other species, this region was reported to be part of ycf2 gene as in* C. annuum* also. These observations indicate the presence of duplicate copy of trnH gene sequence in* C. annuum* and two* alternative tRNA* genes coding for proline amino acid in* D. stramonium.* However, no other evidence was found in databases about this particular region coding for trnH.
- Rps19 pseudogene was reported in three species, namely,* N. tomentosiformis*,* S. bulbocastanum*, and* S. tuberosum*. All other species were mined for similar pseudogene using BLAST pairwise algorithm which confirmed the presence of rps19 pseudogene in other species, namely,* A. belladonna*,* C. annuum*, and* D. stramonium*. The presence of pseudogene may be attributed to the expansion of IRB into the LSC region. In three species, namely,* N. sylvestris*,* N. tabacum*, and* N. undulata*, rps19 pseudogene was found to be absent.
- infA, a pseudogene for all species except* A. belladonna*,* D. stramonium*, and* S. Lycopercicum*, is a protein-coding gene for* S. bulbocastanum*. Homology search with infA sequence from* S. bulbocastanum* against plastomes of* A. belladonna* and* D. stramonium* revealed identical sequence in both species.
- sprA gene has been annotated for* N. sylvestris*,* N. tomentosiformis*,* S. lycopersicum*, and* S. tuberosum*. Its identical orthologous gene sequences were found in* A. belladonna*,* C. annuum*,* D. stramonium*,* N. tabacum*,* N. undulata*, and* S. bulbocastanum* using BLAST search.

### 3.3. Split Genes

A total of eighteen split genes have been reported. The sizes of exons and introns for these genes in all the solanaceous species studied are summarized in Table 2. The rps12 gene is divided such that its 5′ end exon is located in the LSC region whereas second and third exons are located in the IR region. Maturation of RNA transcript requires a trans-splicing mechanism between exon 1 and exon 2 [34, 37]. Among the eighteen intron-containing genes, ycf3, clpP, and rps12 contained two introns whereas the other 15 genes contain only one intron. As per Kim and Lee [34] trnL-UAA gene intron belongs to the self-splicing group I intron whereas all other introns belong to group II. Generally, the size of exons was shown to be conserved and variability was observed in the intron regions; however, ndhB was found to be highly conserved for both exons and introns.

### 3.4. Pairwise Comparison of Plastid Genes of Solanaceae and InDel Analyses

Pairwise comparison of nucleotide sequences of individual gene sequences (45 combinations) for 116 genes was also performed to classify genes based on percent identity. Supplementary Table 1 (Supplementary Material available online at http://dx.doi.org/10.1155/2014/424873) shows grouping of genes in different clusters based on percent identity in pairwise comparison. Genes which showed 100% identity in comparison were considered as highly conserved and the genes showing less than 95% identity at least once in the comparison were considered as highly divergent. These highly divergent genes were further explored at nucleotide as well as at protein level to probe the variations in detail. A total of 11 highly divergent genes were found whereas the number of highly conserved genes varied from 26 (for species pair:* N. tomentosiformis* and* S. lycopersicum*) to 107 (for species pair:* N. sylvestris* and* N. tabacum*). Most of the tRNA genes were found to be highly conserved. Genes accD, cemA, clpP, ndhA, rpl32, rpl36, rps16, sprA, trnA-UGC, trnL-UAA, and ycf1 were found to be highly diverged.

**Figure 1:**
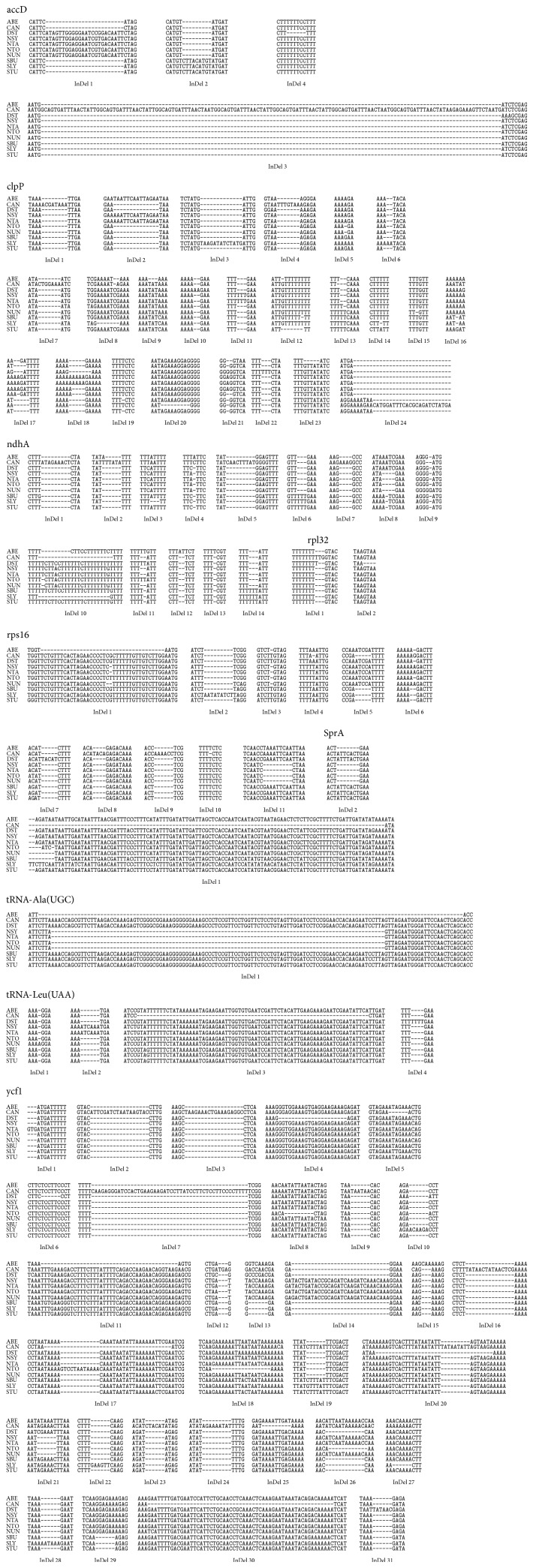
Partial multiple sequence alignment of accD, clpP, ndhA, rpl32, rps16, sprA, tRNA-Ala (UGC), tRNA-Leu(UAA), and ycf1 gene sequences of ten solanaceous species showing location of InDels indicated by hyphens.

**Figure 2:**
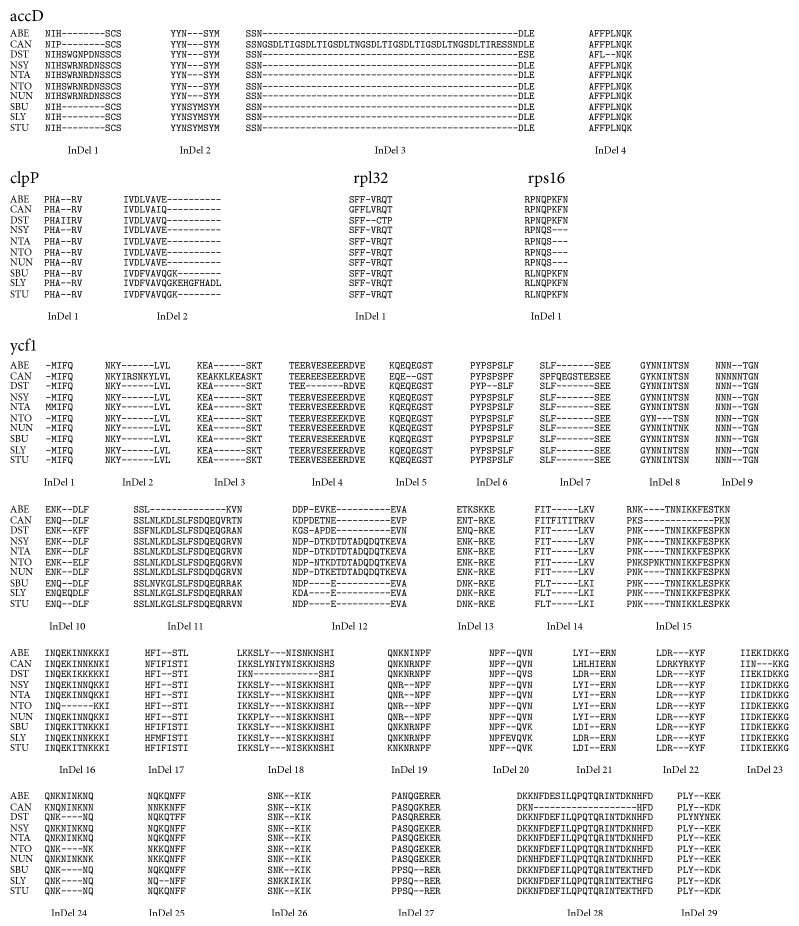
Partial multiple sequence alignment of amino acid sequences of genes, namely, accD, clpP, ndhA, rpl32, rps16, sprA, tRNA-Ala(UGC), tRNA-Leu(UAA), and ycf1, of ten solanaceous species showing location of InDels indicated by hyphens.

Tables 3 and 4 describe the summary of InDels observed in nucleotide and amino acid sequences, respectively. Partial multiple sequence alignment of 9 genes and 5 proteins is shown in Figures 1 and 2, respectively. The longest insertion of 141 bp was observed in accD gene sequence of* C. annuum.* Since genes clpP, ndhA, rps16, and trnL-UAA contained introns, it was important to examine whether these InDels were present in exon or intron region. It was found that all the InDels reported in ndhA and trnL-UAA were present in introns whereas, in case of clpP, InDel 24 was located in exon of the gene. Similarly, the first and last InDels of gene rps16 were present in exons of the gene. Keeping in view the observations in number and length of InDels in nucleotide and protein sequences of genes under consideration, the variation for individual genes is discussed below.

*(1) accD*. A total of four InDels were observed in accD gene as depicted in Figure 1. Insertion of 24 bp was present interestingly in all* Nicotiana* species and* D. stramonium* followed by insertion of 9 bp in all* Solanum* species indicating stronger sequence conservation at genus level. These insertions were also reported by Chung et al. [25]. A 141 bp insertion was observed specifically in* C. annuum* which has also been reported by Jo et al. [29] and confirmed by RT-PCR. Similarly a species specific deletion of 6 bp was found in* D. stramonium*. All these InDels were also reflected in the corresponding protein sequences as shown in Figure 2. The accD gene has been reported to be one of the most variable plastid genes and is probably under diversifying selection [26].

*(2) clpP*. In clpP gene InDels were found both in intron and in exon regions. Two major consequences were observed in the InDels in the exon regions. An insertion of 6 bp in* S. bulbocastanum* and* S. tuberosum* and 30 bp in* S. lycopersicum* at 3′ end (exon 3) of the clpP gene resulted in shifting of stop codon by 6, 6, and 30 bp downstream in respective species compared to other species of Solanaceae family, increasing the length of the coding sequence and the protein product (Figures 1 and 2). An interesting feature was observed as InDel 1 in protein sequence corresponding to insertion of a repeat of “I” amino acids in* D. stramonium* making exon 3 region longer by 6 bp. This region however corresponds to the end of intron 2 in clpP gene in all other species. Since* D. stramonium* chloroplast genome has been sequenced recently, this observation needs to be experimentally validated.

*(3) ndhA*. All the InDels found in ndhA were present in introns while the protein-coding regions (exons) were highly conserved. This indicates high diversifying selection on intronic region of this gene. Out of the total 14 InDels most of the InDels were observed with respect to* C. annuum* (InDels 1, 2, 5, 7, and 10). InDel 10 was observed to be shared by* C. annuum* and* S. lycopersicum* in full and by* C. annuum* and* A. belladonna* in part.

*(4) rpl32*. In rpl32 insertion of 1 bp in* D. stramonium* and 3 bp in* C. annuum* was found in the 3′ region of gene while a deletion of 4 bp was observed in* D. stramonium*. The insertion of 3 bp in* C. annuum* only altered the length of the protein by making it longer by 1 amino acid. However, the small insertion of 1 bp in* D. stramonium* proved to be a frameshift mutation resulting in three changes in the amino acid sequence near the C-terminus. Moreover, deletion of 4 bp at the 3′ end resulted in premature termination of protein synthesis. The frameshift mutation and the 3′ end deletion finally reduced the gene product length by 1 amino acid. As the C-terminal of amino acid chain is well conserved in all the other species, the effect of above mentioned variations needs to be validated experimentally.

*(5) rps16*. In rps16 also InDels were observed in introns as well as exons. Five of the major insertions in the intron regions were species specific. Insertion of 38 bp (InDel 1), 9 bp (InDel 2), 5 bp (InDel 7), 4 bp (InDel 8), and 6 bp (InDel 9) was observed in* A. belladonna*,* S. lycopersicum*,* D. stramonium*, and* C. annuum*. A deletion of 5 bp was observed in all the three* Solanum* species and* C. annuum*. A deletion of 9 bp was observed in all* Nicotiana* species resulting in an amino acid change (P to S) and shortening of protein by three amino acids in the C-terminal region. Similar deletion has also been observed by Kahlau et al. [27] and was suggested to be functionally neutral.

*(6) sprA*. sprA gene has been reported as stable noncoding RNA of unknown function. This gene has been suggested to influence 16S rRNA maturation [38, 39]. In many species this gene seems to be present as remnant and shows large variations in its 5′ region. The largest deletion of 109 bp was observed in* C. annuum*. The rest of this gene appears to be more conserved with a deletion towards the 3′ end in all* Nicotiana* species and* A. belladonna*. The manner in which this gene functions and the consequences of the above mentioned variations are yet to be investigated experimentally.

*(7) trnA-UGC*. In this particular gene a long deletion of 102 bp was observed in all* Nicotiana* species. Interestingly, this deletion was further extended to 130 bp in both directions in* A. belladonna*. These deletions were found in the intron region and so are unlikely to have any negative impact on gene product function.

*(8) trnL-UAA*. The trend of variation in trnL-UAA was similar to that in ndhA as all InDels were observed in introns. The longest species specific deletion (InDel 3) was observed in* C. annuum* whereas short insertion of four nucleotides, a repeat of “T,” was observed specifically in* D. stramonium*. Another insertion of 6 bp was observed in two* Nicotiana* species, that is,* N. sylvestris* and* N. tabacum*.

*(9) ycf1*. Many InDels (3′ region) were found in the fastest evolving gene, that is, ycf1 gene. Most of the InDels were found to be species specific. Maximum InDels (InDels 2, 3, 5, 7, 9, 16, 17, 19, 23, 24, 25, and 30) were observed in* C. annuum* followed by* D. stramonium* (InDels 4, 6, 20, 26, and 31), by* N. tomentosiformis* (InDels 8, 17, and 18), and by* S. lycopersicum* (InDels 16, 22, and 28). Two genus specific InDels (InDels 14 and 21) were observed in all the four* Nicotiana* species. However, InDel 19 was also present in* D. stramonium*. Another genus specific InDel (InDel 29) was observed in* Solanum* species. Most of the InDels altered the length of the gene product with maximum length of 1906 amino acids (aa) observed in* C. annuum* and the minimum of 1873 aa observed in* D. stramonium*. Among the* Solanum* species the length of protein (1887 aa) was conserved among* S. bulbocastanum* and* S. tuberosum*. However,* S. lycopersicum* was having the amino acid sequence of 1891 aa, larger by 4 aa as compared to the other two species of the same genus. Among the four* Nicotiana* species the ycf1 gene product length was conserved among* N. sylvestris*,* N. tabacum*, and* N. undulata* having protein lengths of 1901 aa, 1902 aa, and 1901 aa, respectively. However,* N. tomentosiformis* was observed to be the most variable member of the genus* Nicotiana* having protein length of 1892 aa.

### 3.5. Phylogenetic Analysis of Solanaceous Plastomes

Evolutionary relationships between diverse plant species have been analyzed using several plastome markers including matK and rbcL (genes) or trnH-psbA and trnL-trnF (intergenic regions) due to sequence conservation among plant taxa blended with suitable variation [40, 41]. However, determination of phylogeny based on single gene sequences may be inaccurate [42]. Availability of complete chloroplast sequences for many species has made it possible to use many individual genes or concatenated gene sequences to deduce phylogenetic relationships among taxa [42–44].

**Figure 3:**
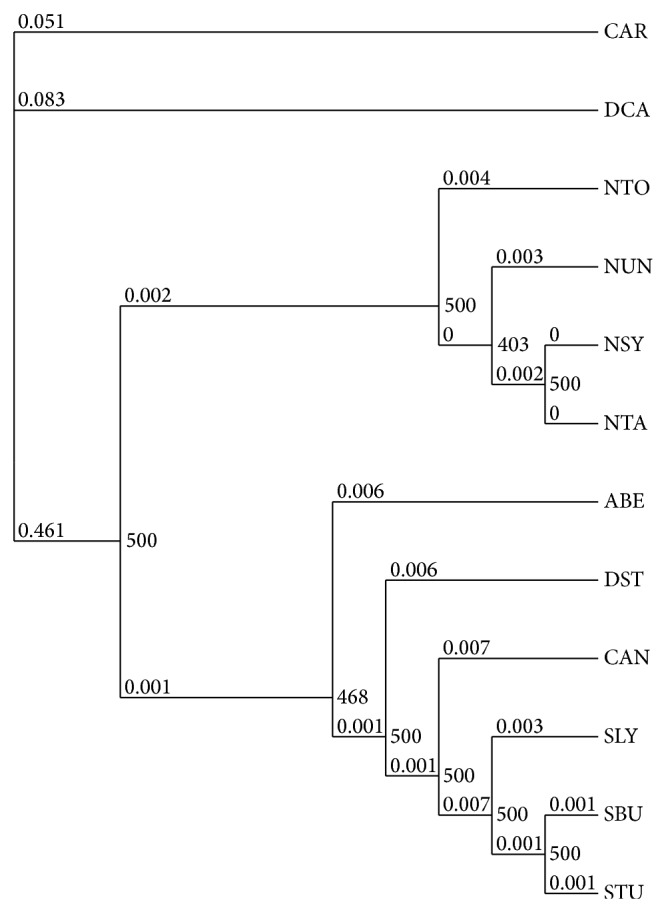
Maximum likelihood phylogenetic tree derived using concatenated nucleotide sequences of 75 protein-coding genes of ten solanaceous species and two outgroup species.

Efforts have been made to carry out phylogenetic analysis of solanaceous species using complete plastome sequences by Moore et al. [44] and Jansen et al. [45]. Evolutionary positions of* Capsicum* and* Datura* in Solanaceae have been determined using a single or a few plastid genes [46, 47]. Recently, concatenated protein-coding gene sequences from completely sequenced plastomes were used to obtain reasonable phylogenetic relationships for solanaceous species [29]. In the present investigation we also used a similar approach to analyze the phylogenetic relationship for ten solanaceous species with completely sequenced plastomes. Individual multiple sequence alignments were concatenated for maximum likelihood phylogenetic tree generation. As depicted in Figure 3, taxa were divided into two clades with 100% bootstrap value of 500. The first clade consisted of four* Nicotiana* species while the species in* Solanum*,* Capsicum*,* Atropa*, and* Datura* were included in the second clade. These results are in line with previous phylogenetic analyses using concatenated protein-coding gene sequences as well as phylogenetic analyses using plastid ndhF and trnL-F sequences [29, 47]. However, in an analysis of 13 orfs of solanaceous plastomes, a different arrangement was shown in which* Atropa* was shown to be separated from* Solanum* and was grouped together with* Nicotiana* [25].

## 4. Conclusions

The analyses of complete plastid genomes of ten solanaceous species revealed overall similarity in terms of the gene content and organization. The sizes of LSC, SSC, and IR regions were found to be somewhat conserved among species but a significant variation was found between genera. Most of the coding regions were well conserved. However, many of the features in few genes were observed to be typical of a particular genus and even species, which can be used as molecular markers to investigate genetic diversity and evolution. These typical variations can be further utilized to develop more sophisticated DNA barcoding based techniques. Ten solanaceous species are divided into two clades on the basis of Phylogenetic analysis using concatenated alignment of gene sequences from coding regions of plastomes. The first clade consisted of four* Nicotiana* species and the second clade consisted of species of* Solanum*,* Capsicum*,* Atropa*, and* Datura*.

## Supplementary Material

Supplementary table 1: shows grouping of genes in different clusters based on percent identity in pairwise comparison. Ten clusters were made depending upon percentage identity observed between the genes ranging from 80% (minimum identity observed for a given gene between any two species) to 100%. Genes which showed 100% identity in comparison were considered as highly conserved and the genes showing less than 95% identity at least once in the comparison were considered highly divergent. These highly divergent genes were further explored at nucleotide as well as at protein level to probe the variations in detail. A total of 11 highly divergent genes were found whereas the number of highly conserved genes varied from 26 (for species pair: *N. tomentosiformis* and *S. lycopersicum*) to 107 (for *N. sylvestris* and *N. tabacum*). Most of the tRNA genes were found to be highly conserved. Genes accD, cemA, clpP, ndhA, rpl32, rpl36, rps16, sprA, trnA-UGC, trnL-UAA and ycf1 were found to be highly diverged.

## Figures and Tables

**Table 1:** Properties of the solanaceous chloroplast genomes.

Property	Name of species									
	ABE	CAN	DST	NSY	NTA	NTO	NUN	SBU	SLY	STU
Genome size (bp)	156687	156781	155871	155941	155943	155745	155863	155371	155461	155296
LSC (bp) (coordinates)^*^	86,869 (1–86869)	87366 (1–87366)	86297 (1–86297)	86684 (1–86684)	86,686 (1–86686)	86392 (1–86392)	86633 (1–86633)	85785 (1–85785)	85,882 (1–85882)	85737 (1–85737)
IR_B_ (bp) (coordinates)^*^	25,905 (86870–112774)	25783 (87367–113149)	25563 (86298–111860)	25342 (86685–112026)	25,343 (86687–112029)	25429 (86393–111821)	25331 (86634–111964)	25588 (85786–111373)	25,608 (85883–111490)	25593 (85738–111330)
SSC (bp) (coordinates)^*^	18,008 (112775–130782)	17849 (113150–130998)	18448 (111861–130308)	18573 (112027–130599)	18,571 (112030–130600)	18495 (111822–130316)	18568 (111965–130532)	18381 (111374–129754)	18,363 (111491–129853)	18373 (111331–129703)
IR_A_ (bp) (coordinates)^*^	25,905 (130783–156687)	25783 (130999–156781)	25563 (130309–155871)	25342 (130600–155941)	25,343 (130601–155943)	25429 (130317–155745)	25331 (130533–155863)	25588 (129755–155342)	25,608 (129854–155461)	25593 (129704–155296)
Coding regions (%)	58.89	58.50	59.19	61.49	61.12	61.58	63.12	58.52	58.91	58.45
Introns (%)	12.51	12.71	11.62	12.70	12.70	12.68	12.69	12.82	12.47	12.49
Intergenic regions (%)	28.60	28.79	29.19	25.81	26.18	25.73	24.19	28.66	28.62	29.06

AT content (%)										
Overall	62.44	62.27	62.12	62.15	62.15	62.21	62.12	62.12	62.14	62.12
Coding regions	59.86	59.68	59.65	59.85	59.79	59.79	59.70	59.61	59.65	59.59
Noncoding regions	66.13	65.93	65.72	65.84	65.87	66.09	66.27	65.66	65.71	65.68
tRNAs	47.70	47.38	47.08	47.06	47.05	47.10	47.08	47.12	47.01	47.06
rRNAs	44.64	44.73	44.63	44.64	44.64	44.64	44.64	44.66	44.66	44.65
Protein-coding genes	62.01	61.83	61.79	61.91	61.86	61.84	61.68	61.76	61.80	61.74
LSC	64.37	64.25	64.04	64.05	64.05	64.12	64.01	63.99	64.01	63.99
SSC	68.35	67.99	67.72	67.94	67.93	68.03	67.87	67.87	67.97	67.91
IR	57.14	56.94	56.87	56.78	56.78	56.84	56.78	56.93	56.91	56.90

ABE: *Atropa belladonna*, CAN: *Capsicum annuum*, DST: *Datura stramonium*, NSY: *Nicotiana sylvestris*, NTA: *Nicotiana tabacum*, NTO: *Nicotiana tomentosiformis*, NUN: *Nicotiana undulata*, SBU: *Solanum bulbocastanum*, SLY: *Solanum lycopersicum*, STU: *Solanum tuberosum*, LSC: large single copy region, SSC: small single copy region, and IR: inverted repeat region.

^*^Start and end position of nucleotide in the genome.

**Table 2:** The lengths of introns and exons for the split genes of ten solanaceous species.

Gene (region)	Exon/intron	ABE	CAN	DST	NSY	NTA	NTO	NUN	SBU	SLY	STU
trnK-UUU (LSC)	Exon I	37	37	37	37	37	37	37	37	37	37
	Intron I	2519	2500	2506	2526	2526	2526	2521	2501	2514	2512
	Exon II	36	35	35	35	35	35	35	35	35	35

rps16 (LSC)	Exon I	40	40	40	40	40	40	40	40	40	40
	Intron I	822	865	866	860	860	860	859	855	864	855
	Exon II	227	227	227	218	218	218	218	227	227	227

trnG-UCC (LSC)	Exon I	23	23	23	23	23	23	23	23	23	23
	Intron I	692	692	694	692	692	690	691	701	695	692
	Exon II	48	48	48	48	48	48	48	37	48	48

atpF (LSC)	Exon I	145	145	145	145	145	145	145	144	144	145
	Intron I	715	693	700	695	695	692	692	693	686	693
	Exon II	410	410	410	410	410	410	410	411	411	410

rpoC1 (LSC)	Exon I	432	453	453	453	453	432	453	453	453	453
	Intron I	737	742	737	737	737	709	733	737	737	737
	Exon II	1614	1614	1614	1614	1614	1614	1623	1614	1614	1614

ycf3 (LSC)	Exon I	124	124	124	124	124	124	124	124	124	124
	Intron I	739	742	740	739	738	731	735	730	729	727
	Exon II	230	230	230	230	230	230	230	230	230	230
	Intron II	763	744	753	783	783	779	781	750	750	750
	Exon III	153	153	159	153	153	153	153	153	153	153

trnL-UAA (LSC)	Exon I	35	35	35	35	35	35	35	37	35	35
	Intron I	497	426	501	503	503	497	498	502	497	497
	Exon II	50	50	50	50	50	50	50	50	50	50

trnV-UAC (LSC)	Exon I	38	38	38	38	38	38	38	38	38	38
	Intron I	572	575	569	571	571	572	573	569	571	571
	Exon II	35	35	37	35	35	35	35	37	35	35

rps12^*^	Exon I	114	114	114	114	114	114	114	114	114	114
	Intron I	—	—	—	—	—	—	—	—	—	—
	Exon II	232	232	232	232	232	232	232	232	232	232
	Intron II	535	536	536	536	536	536	536	536	536	536
	Exon III	26	26	26	26	26	26	26	26	26	26

clpP (LSC)	Exon I	71	71	71	71	71	71	71	71	71	71
	Intron I	799	811	792	807	807	789	789	789	798	789
	Exon II	292	292	292	292	292	292	292	292	292	292
	Intron II	622	626	624	637	637	634	631	625	617	620
	Exon III	228	228	234	228	228	228	228	234	258	234

petB (LSC)	Exon I	6	6	6	6	6	6	6	6	6	6
	Intron I	759	755	746	753	753	753	753	747	747	747
	Exon II	642	642	642	642	642	642	642	642	642	642

petD (LSC)	Exon I	8	8	9	8	8	8	8	6	8	8
	Intron I	742	742	748	742	742	742	742	739	738	739
	Exon II	475	475	474	475	475	475	475	477	475	475

rpl16 (LSC)	Exon I	9	9	9	9	9	9	9	9	9	9
	Intron I	1019	1026	1025	1020	1020	1021	1020	1014	1018	1014
	Exon II	396	396	396	396	396	396	396	396	396	396

rpl2 (IR)	Exon I	391	391	393	391	391	391	391	390	391	391
	Intron I	664	665	669	666	666	666	666	666	666	666
	Exon II	434	434	429	434	434	434	434	435	434	434

ndhB (IR)	Exon I	777	777	777	777	777	777	777	777	777	777
	Intron I	679	679	679	679	679	679	679	679	679	679
	Exon II	756	756	756	756	756	756	756	756	756	756

trnI-GAU (IR)	Exon I	37	37	42	37	37	37	37	42	37	37
	Intron I	717	722	717	707	707	716	716	717	722	722
	Exon II	34	35	35	35	35	35	35	35	35	35

trnA-UGC (IR)	Exon I	38	38	38	38	38	38	38	38	38	38
	Intron I	681	811	811	709	709	709	709	811	811	811
	Exon II	35	35	35	35	35	35	35	35	35	35

ndhA (SSC)	Exon I	553	553	552	553	553	553	553	552	553	553
	Intron I	1150	1157	1154	1148	1148	1149	1148	1158	1133	1158
	Exon II	539	539	537	539	539	539	539	540	539	539

^*^rps12 gene is dividing gene. The 3′-rps12 locates on the IR-region, while the 5′-rps12 locates on the LSC region.

ABE: *Atropa belladonna*, CAN: *Capsicum annuum*, DST: *Datura stramonium*, NSY: *Nicotiana sylvestris*, NTA: *Nicotiana tabacum*, NTO: *Nicotiana tomentosiformis*, NUN: *Nicotiana undulata*, SBU: *Solanum bulbocastanum*, SLY: *Solanum lycopersicum*, and STU: *Solanum tuberosum.*

**Table 3:** InDels in nucleotide sequences of 9 genes of ten solanaceous plastid genomes.

S. number	Gene^abc^	Total number of InDels	InDel length (bp)
1	accD^a^	4	24, 9, 141, 6
2	clpP^a^	24	8(I), 14(I), 13(I), 7(I), 1(I), 2-3(I), 7(I), 1–7(I), 3(I), 2(I), 3(I), 1–7(I), 1–3(I), 1(I), 1(I), 1(I), 1–5(I), 4–7(I), 1(I), 9(I), 1-2(I), 3(I), 5(I), 24–30
3	ndhA^b^	14	9(I), 5-6(I), 3(I), 1(I), 9(I), 3(I), 4(I), 1–4(I), 1-2(I), 1–23(I), 1-2(I), 2(I), 1(I), 3(I)
4	rpl32^b^	2	2-3, 4
5	rps16^a^	11	1–38, 9(I), 1(I), 1(I), 5(I), 1-2(I), 5(I), 4(I), 6(I), 1(I), 9
6	sprA^b^	2	109, 7
7	trnA-UGC^c^	1	102–130
8	trnL-UAA^a^	4	1, 6, 71, 4
9	ycf1^b^	31	3, 18, 18, 21, 6, 6, 48, 9, 6, 6, 42, 3, 6, 30, 3, 15, 12–39, 18, 6, 9–36, 6, 6, 6, 9, 9, 12, 6, 6, 6, 57, 6

^abc^Location in different regions; ^a^LSC, ^b^SSC, and ^c^IR; I: InDels present in introns.

**Table 4:** InDels in amino acid sequences of 5 proteins of ten solanaceous plastid genomes.

S. number	Protein	Total number of InDels	InDel length (bp)
1	accD	4	8, 3, 47, 2
2	clpP	2	2, 10
3	rpl32	1	1-2
4	rps16	1	3
5	ycf1	29	1, 6, 6, 7, 2, 2, 7, 3, 2, 2, 14, 1–10, 1, 5, 4–13, 6, 2, 3–12, 2, 2, 2, 3, 3, 4, 2, 2, 2, 19, 2
