# Supplementary material for: Comparative Genomics of Ten Solanaceous Plastomes
Source: Adv Bioinformatics. 2014 Nov 17;2014:424873. doi: 10.1155/2014/424873 (PMC4248371; doi:10.1155/2014/424873)
Supplement: Supplementary file 1 — Supplementary table 1: shows grouping of genes in different clusters based on percent identity in pairwise comparison. Ten clusters were made depending upon percentage identity observed between the genes ranging from 80% (minimum identity observed for a given gene between any two species) to 100%. Genes which showed 100% identity in comparison were considered as highly conserved and the genes showing less than 95% identity at least once in the comparison were considered highly divergent. These highly divergent genes were further explored at nucleotide as well as at protein level to probe the variations in detail. A total of 11 highly divergent genes were found whereas the number of highly conserved genes varied from 26 (for species pair: N. tomentosiformis and S. lycopersicum) to 107 (for N. sylvestris and N. tabacum). Most of the tRNA genes were found to be highly conserved. Genes accD, cemA, clpP, ndhA, rpl32, rpl36, rps16, sprA, trnA-UGC, trnL-UAA and ycf1 were found to be highly diverged. [file 424873.f1.doc]

**Table 1.** Grouping of chloroplast genes in identity classes based on pairwise comparison.

| **Species pair** | **Identity (%)** | | | | | | | | | |
| --- | --- | --- | --- | --- | --- | --- | --- | --- | --- | --- |
|  | **100%** | **99 - 99.99 %** | **98-98.99%** | **97-97.99%** | **95-96.99%** | **93-94.99%** | **91-92.99%** | **88-90.99%** | **85-87.99%** | **80-84.99%** |
| **ABE-CAN** | petG, petL, petN, psbF, psbJ, psbK, psbM, psbN, psbZ, rps19, rrn4.5, tRNA-Arg(ACG), tRNA-Arg(UCU), tRNA-Asn(GUU), tRNA-Cys(GCA), tRNA-fM(CAU), tRNA-Glu(UUC), tRNA-His(GUG), tRNA-Ile(CAU), tRNA-Leu(CAA), tRNA-Met(CAU), tRNA-Phe(GAA), tRNA-Ser(GCU), tRNA-Ser(UGA), tRNA-Thr(GGU), tRNA-Thr(UGU), tRNA-Trp(CCA), tRNA-Val(GAC), tRNA-Gly(GCC) (29) | atpI, ndhB, ndhJ, psaA, psaB, psaI, psaJ, psbA, psbB, psbD, psbI, psbL, psbT, rpl2, rpl23, rps2, rps7, rps11, rps12, rps14, rps18, rrn5, rrn16, tRNA-Ile(GAU), ycf4 | atpA, atpB, atpE, atpH, ndhC, ndhE, ndhG, ndhH, ndhI, ndhK, petA, petD, psaC, psbC, psbE, psbH, rbcL, rpl14, rpl33, rpoA, rpoB, rpoC1, rpoC2, rps3, rps4, rps15, rrn23, tRNA-Asp(GUC), tRNA-Gln(UUG), tRNA-Leu(UAG), tRNA-Pro(UGG), tRNA-Ser(GGA), tRNA-Tyr(GUA), ycf2 | ccsA, cemA, matK, ndhD, ndhF, petB, rpl16, rpl22, rpl36, rps8, tRNA-Val(UAC), tRNA-Gly(UCC), ycf3 | atpF, ndhA, rpl20, tRNA-Lys(UUU) | clpP, rps16 |  | accD, rpl32, ycf1 | tRNA-Leu (UAA) | tRNA-Ala (UGC) |
| **ABE-DST** | petG, petL, petN, psaI, psbI, psbJ, psbM, psbN, rps7, rrn4.5, rrn5, tRNA-Arg(ACG), tRNA-Asn(GUU), tRNA-Cys(GCA), tRNA-fM(CAU), tRNA-Gln(UUG), tRNA-Glu(UUC), tRNA-His(GUG), tRNA-Ile(CAU), tRNA-Leu(CAA), tRNA-Met(CAU), tRNA-Phe(GAA), tRNA-Ser(GCU), tRNA-Ser(UGA), tRNA-Thr(GGU), tRNA-Thr(UGU), tRNA-Trp(CCA), tRNA-Val(GAC), tRNA-Gly(GCC) (29) | atpB, atpE, atpH, atpI, ndhB, ndhJ, psaA, psaB, psaJ, psbA, psbB, psbC, psbD, psbE, psbF, psbH, psbK, psbL, psbT, psbZ, rpl2, rpl23, rpl33, rpoB, rps2, rps4, rps11, rps12, rps14, rps18, rrn16, rrn23, tRNA-Ile(GAU), ycf4 | atpA, ndhC, ndhE, ndhG, ndhH, ndhI, ndhK, petA, rbcL, rpl14, rpl20, rpl36, rpoA, rpoC1, rpoC2, rps3, rps8, rps19, tRNA-Arg(UCU), tRNA-Leu(UAG), tRNA-Pro(UGG), tRNA-Ser(GGA), tRNA-Tyr(GUA), tRNA-Val(UAC), ycf2 | atpF, ccsA, cemA, matK, ndhA, ndhD, ndhF, petD, psaC, rps15, tRNA-Leu(UAA), tRNA-Lys(UUU), tRNA-Gly(UCC), ycf3 | accD, clpP, petB, rpl16, rpl22, rpl32 | rps16, ycf1 | sprA |  | tRNA-Ala (UGC) |  |
| **ABE-NSY** | petG, petL, psbF, psbI, psbM, psbN, psbZ, rpl23, rps7, rrn4.5, rrn5, tRNA-Arg(ACG), tRNA-Arg(UCU), tRNA-Asn(GUU), tRNA-Cys(GCA), tRNA-fM(CAU), tRNA-Gln(UUG), tRNA-Glu(UUC), tRNA-His(GUG), tRNA-Ile(CAU), tRNA-Met(CAU), tRNA-Phe(GAA), tRNA-Ser(GCU), tRNA-Ser(GGA), tRNA-Thr(GGU), tRNA-Thr(UGU), tRNA-Trp(CCA), tRNA-Val(GAC), tRNA-Gly(GCC) (29) | atpB, atpI, ndhB, ndhJ, psaA, psaB, psaI, psaJ, psbA, psbB, psbC, psbD, psbJ, psbL, psbT, rbcL, rpl2, rpl20, rpl33, rpl36, rpoB, rps2, rps4, rps11, rps18, rps19, rrn16, rrn23, ycf2, ycf4 | atpA, atpE, atpH, cemA, matK, ndhC, ndhE, ndhF, ndhG, ndhH, ndhI, ndhK, petA, petB, petD, petN, psaC, psbE, psbH, psbK, rpl14, rpl16, rpoA, rpoC1, rpoC2, rps3, rps8, rps14, tRNA-Asp(GUC), tRNA-Ile(GAU), tRNA-Leu(CAA), tRNA-Leu(UAG), tRNA-Tyr(GUA), tRNA-Val(UAC), tRNA-Gly(UCC) | ccsA, clpP, ndhD, rpl22, rpl32, rps12, rps15, tRNA-Leu(UAA), tRNA-Pro(UGG), tRNA-Ser(UGA) | accD, atpF, ndhA, sprA, tRNA-Ala(UGC), tRNA-Lys(UUU), ycf1, ycf3 | rps16 |  |  |  |  |
| **ABE-NTO** | petG, petL, psbF, psbI, psbJ, psbM, psbN, psbZ, rpl23, rps7, rrn4.5, rrn5, tRNA-Arg(ACG), tRNA-Asn(GUU), tRNA-Cys(GCA), tRNA-fM(CAU), tRNA-Gln(UUG), tRNA-Glu(UUC), tRNA-His(GUG), tRNA-Ile(CAU), tRNA-Met(CAU), tRNA-Phe(GAA), tRNA-Ser(GGA), tRNA-Thr(GGU), tRNA-Thr(UGU), tRNA-Trp(CCA), tRNA-Val(GAC), tRNA-Gly(GCC) (28) | atpI, ndhB, ndhG, ndhJ, psaA, psaB, psaI, psbA, psbB, psbD, psbK, psbL, rbcL, rpl2, rpl20, rpl33, rpoB, rps2, rps4, rps11, rps18, rps19, rrn16, rrn23, tRNA-Ile(GAU), tRNA-Leu(UAA), ycf2, ycf4 | atpA, atpB, atpH, cemA, ndhC, ndhE, ndhI, ndhK, petA, petD, psaC, psaJ, psbC, psbE, psbH, psbT, rpl14, rpl36, rpoA, rpoC2, rps3, rps8, rps12, rps14, tRNA-Arg(UCU), tRNA-Asp(GUC), tRNA-Leu(CAA), tRNA-Leu(UAG), tRNA-Ser(GCU), tRNA-Tyr(GUA), tRNA-Val(UAC), tRNA-Gly(UCC) | atpE, ccsA, matK, ndhD, ndhF, ndhH, petB, petN, rpl16, rpl32, rpoC1, rps15, tRNA-Pro(UGG), tRNA-Ser(UGA) | accD, atpF, clpP, ndhA, rpl22, sprA, tRNA-Ala(UGC), tRNA-Lys(UUU), ycf3 | rps16, ycf1 |  |  |  |  |
| **ABE-NUN** | petG, petL, psbF, psbI, psbJ, psbM, psbN, psbZ, rpl23, rps7, rps19, rrn4.5, rrn5, tRNA-Arg(ACG), tRNA-Arg(UCU), tRNA-Asn(GUU), tRNA-Cys(GCA), tRNA-fM(CAU), tRNA-Gln(UUG), tRNA-Glu(UUC), tRNA-His(GUG), tRNA-Ile(CAU), tRNA-Met(CAU), tRNA-Phe(GAA), tRNA-Ser(GCU), tRNA-Ser(GGA), tRNA-Thr(GGU), tRNA-Thr(UGU), tRNA-Trp(CCA), tRNA-Val(GAC), tRNA-Gly(GCC) (31) | atpB, atpI, cemA, ndhB, ndhJ, psaA, psaB, psaI, psaJ, psbA, psbB, psbC, psbD, psbK, psbL, psbT, rpl2, rpl20, rpl33, rpl36, rpoB, rps2, rps4, rps11, rps12, rps18, rrn16, rrn23, tRNA-Ile(GAU), ycf2, ycf4 | atpA, atpE, atpH, matK, ndhC, ndhE, ndhF, ndhG, ndhH, ndhI, ndhK, petA, petD, petN, psaC, psbE, psbH, rbcL, rpl14, rpoA, rpoC1, rpoC2, rps3, rps8, rps14, tRNA-Asp(GUC), tRNA-Leu(CAA), tRNA-Leu(UAA), tRNA-Leu(UAG), tRNA-Tyr(GUA), tRNA-Val(UAC), tRNA-Gly(UCC) | accD, ndhA, ndhD, petB, rpl16, rpl22, rpl32, rps15, tRNA-Pro(UGG), tRNA-Ser(UGA), ycf3 | atpF, ccsA, clpP, sprA, tRNA-Ala(UGC), tRNA-Lys(UUU), ycf1 | rps16 |  |  |  |  |
| **ABE-SBU** | petG, petL, petN, psaI, psbF, psbI, psbJ, psbK, psbN, psbZ, rpl23, rps7, rps19, rrn4.5, rrn5, tRNA-Arg(ACG), tRNA-Arg(UCU), tRNA-Asn(GUU), tRNA-Cys(GCA), tRNA-fM(CAU), tRNA-Gln(UUG), tRNA-Glu(UUC), tRNA-His(GUG), tRNA-Ile(CAU), tRNA-Leu(CAA), tRNA-Met(CAU), tRNA-Ser(GCU), tRNA-Ser(UGA), tRNA-Thr(GGU), tRNA-Thr(UGU), tRNA-Trp(CCA), tRNA-Val(GAC), tRNA-Gly(GCC) (33) | atpB, atpI, ndhB, psaA, psaB, psaJ, psbA, psbB, psbD, psbL, psbM, psbT, rpl2, rpl20, rps2, rps12, rps14, rps18, rrn16, rrn23, tRNA-Ile(GAU) | atpA, atpE, atpH, ndhC, ndhE, ndhH, ndhI, ndhJ, ndhK, petA, psaC, psbC, psbE, psbH, rbcL, rpl14, rpl32, rpl33, rpoA, rpoB, rpoC1, rpoC2, rps3, rps4, rps8, rps11, rps15, tRNA-Asp(GUC), tRNA-Leu(UAA), tRNA-Leu(UAG), tRNA-Phe(GAA), tRNA-Pro(UGG), tRNA-Ser(GGA), tRNA-Tyr(GUA), tRNA-Val(UAC), tRNA-Gly(UCC), ycf2, ycf4 | accD, ccsA, matK, ndhD, ndhF, ndhG, petB, petD, rpl16, rpl22, ycf3 | atpF, ndhA, rpl36, tRNA-Lys(UUU) | clpP, rps16, ycf1 | sprA | cemA | tRNA-Ala (UGC) |  |
| **ABE-SLY** | petG, petN, psaI, psbF, psbI, psbJ, psbK, psbN, rpl23, rps7, rps19, rrn4.5, rrn5, tRNA-Arg(ACG), tRNA-Arg(UCU), tRNA-Asn(GUU), tRNA-Cys(GCA), tRNA-fM(CAU), tRNA-Gln(UUG), tRNA-Glu(UUC), tRNA-His(GUG), tRNA-Ile(CAU), tRNA-Leu(CAA), tRNA-Leu(UAG), tRNA-Met(CAU), tRNA-Ser(GCU), tRNA-Ser(UGA), tRNA-Thr(GGU), tRNA-Thr(UGU), tRNA-Trp(CCA), tRNA-Val(GAC), tRNA-Gly(GCC) (32) | atpB, ndhB, ndhI, psaA, psaB, psaJ, psbA, psbB, psbD, psbL, psbM, psbZ, rpl2, rpl20, rps2, rps12, rps14, rps18, rrn16, rrn23, tRNA-Ile(GAU) | atpA, atpE, atpH, atpI, ndhC, ndhE, ndhG, ndhJ, ndhK, petA, petL, psaC, psbC, psbE, rbcL, rpl14, rpl33, rpoA, rpoB, rpoC1, rps3, rps4, rps8, rps11, tRNA-Asp(GUC), tRNA-Leu(UAA), tRNA-Phe(GAA), tRNA-Pro(UGG), tRNA-Ser(GGA), tRNA-Tyr(GUA), tRNA-Val(UAC), ycf2, ycf4 | accD, ndhD, ndhF, ndhH, petB, petD, psbH, psbT, rpl22, rpl32, rpoC2, rps15, tRNA-Gly(UCC), ycf3 | atpF, ccsA, matK, ndhA, rpl16, rpl36, tRNA-Lys(UUU) | clpP, ycf1 | rps16 | cemA, sprA | tRNA-Ala (UGC) |  |
| **ABE-STU** | petG, petL, petN, psaI, psbF, psbJ, psbK, psbN, psbZ, rpl23, rps7, rps19, rrn4.5, rrn5, tRNA-Arg(ACG), tRNA-Arg(UCU), tRNA-Asn(GUU), tRNA-Cys(GCA), tRNA-fM(CAU), tRNA-Gln(UUG), tRNA-Glu(UUC), tRNA-His(GUG), tRNA-Ile(CAU), tRNA-Leu(CAA), tRNA-Met(CAU), tRNA-Ser(GCU), tRNA-Ser(UGA), tRNA-Thr(GGU), tRNA-Thr(UGU), tRNA-Trp(CCA), tRNA-Val(GAC), tRNA-Gly(GCC) (32) | atpB, atpI, ndhB, ndhC, psaA, psaB, psaJ, psbA, psbB, psbD, psbI, psbL, psbM, psbT, rpl2, rpl20, rps2, rps12, rps18, rrn16, rrn23, tRNA-Ile(GAU) | atpA, atpE, atpH, ndhD, ndhE, ndhG, ndhH, ndhI, ndhJ, ndhK, petA, psaC, psbC, psbE, psbH, rpl14, rpl32, rpl33, rpoA, rpoB, rpoC1, rpoC2, rps3, rps4, rps8, rps11, rps14, rps15, tRNA-Asp(GUC), tRNA-Leu(UAA), tRNA-Leu(UAG), tRNA-Phe(GAA), tRNA-Pro(UGG), tRNA-Ser(GGA), tRNA-Tyr(GUA), tRNA-Val(UAC), tRNA-Gly(UCC), ycf2, ycf4 | accD, ccsA, matK, ndhF, petB, petD, rbcL, rpl16, rpl22, ycf3 | atpF, ndhA, rpl36, tRNA-Lys(UUU) | clpP, ycf1 | rps16, sprA | cemA | tRNA-Ala (UGC) |  |
| **CAN-DST** | petG, petL, petN, psaJ, psbJ, psbL, psbM, psbN, psbT, rps12, rrn4.5, rrn16, tRNA-Arg(ACG), tRNA-Asn(GUU), tRNA-Cys(GCA), tRNA-fM(CAU), tRNA-Glu(UUC), tRNA-His(GUG), tRNA-Ile(CAU), tRNA-Ile(GAU), tRNA-Leu(CAA), tRNA-Leu(UAG), tRNA-Met(CAU), tRNA-Phe(GAA), tRNA-Pro(UGG), tRNA-Ser(GCU), tRNA-Ser(GGA), tRNA-Ser(UGA), tRNA-Thr(GGU), tRNA-Thr(UGU), tRNA-Trp(CCA), tRNA-Tyr(GUA), tRNA-Val(GAC), tRNA-Gly(GCC), ycf5 (35) | atpH, atpI, ndhB, ndhC, ndhJ, ndhK, psaA, psaB, psaI, psbA, psbB, psbC, psbD, psbE, psbF, psbI, psbK, psbZ, rpl2, rpl33, rpoB, rps2, rps7, rps11, rrn5, rrn23, tRNA-Ala(UGC), ycf4 | atpA, atpB, atpE, ccsA, ndhD, ndhE, ndhG, ndhH, ndhI, petA, psaC, psbH, rbcL, rpl14, rpl23, rpoA, rpoC1, rpoC2, rps3, rps4, rps8, rps14, rps15, rps18, rps19, tRNA-Arg(UCU), tRNA-Gln(UUG), ycf2 | atpF, matK, ndhF, petB, petD, rpl36, tRNA-Lys(UUU), tRNA-Val(UAC), ycf3 | cemA, clpP, ndhA, rpl16, rpl20, rpl22, rps16, tRNA-Gly(UCC) |  | rpl32 | accD, ycf1 | tRNA-Leu (UAA) |  |
| **CAN-NSY** | petG, petL, psaJ, psbE, psbF, psbL, psbM, psbN, psbT, psbZ, rrn4.5, tRNA-Arg(ACG), tRNA-Arg(UCU), tRNA-Asn(GUU), tRNA-Asp(GUC), tRNA-Cys(GCA), tRNA-fM(CAU), tRNA-Glu(UUC), tRNA-His(GUG), tRNA-Ile(CAU), tRNA-Leu(UAG), tRNA-Met(CAU), tRNA-Phe(GAA), tRNA-Ser(GCU), tRNA-Thr(GGU), tRNA-Thr(UGU), tRNA-Trp(CCA), tRNA-Tyr(GUA), tRNA-Val(GAC), tRNA-Gly(GCC) (30) | atpI, ndhB, ndhE, ndhJ, psaA, psaC, psbA, psbC, psbD, psbI, psbJ, rpl2, rpl23, rpl33, rpoB, rps2, rps7, rps11, rps15, rps18, rps19, rrn5, rrn16, ycf4 | atpA, atpB, atpF, atpH, ccsA, cemA, ndhC, ndhD, ndhG, ndhH, ndhI, ndhK, petA, petB, petD, petN, psaB, psaI, psbB, psbH, psbK, rbcL, rpl14, rpl36, rpoA, rpoC1, rpoC2, rps3, rps4, rps12, rps14, rrn23, tRNA-Gln(UUG), tRNA-Leu(CAA), tRNA-Pro(UGG), tRNA-Ser(GGA), ycf2 | atpE, matK, ndhF, rpl16, rps8, tRNA-Ile(GAU), tRNA-Ser(UGA), tRNA-Val(UAC), tRNA-Gly(UCC) | ndhA, rpl20, rpl22, rps16, tRNA-Lys(UUU), ycf3 | clpP |  | accD, rpl32, tRNA-Ala(UGC), ycf1 | tRNA-Leu (UAA) |  |
| **CAN-NTO** | petG, petL, psbE, psbF, psbJ, psbL, psbM, psbN, psbZ, rrn4.5, tRNA-Arg(ACG), tRNA-Asn(GUU), tRNA-Asp(GUC), tRNA-Cys(GCA), tRNA-fM(CAU), tRNA-Glu(UUC), tRNA-His(GUG), tRNA-Ile(CAU), tRNA-Leu(UAG), tRNA-Met(CAU), tRNA-Phe(GAA), tRNA-Thr(GGU), tRNA-Thr(UGU), tRNA-Trp(CCA), tRNA-Tyr(GUA), tRNA-Val(GAC), tRNA-Gly(GCC) (27) | ndhB, ndhC, ndhE, ndhJ, psaA, psaC, psaJ, psbA, psbD, psbI, psbK, psbT, rpl2, rpl23, rpl33, rpoB, rps2, rps7, rps12, rps18, rps19, rrn5, rrn16, tRNA-Ile(GAU), ycf4 | atpA, atpB, atpF, atpH, atpI, ndhD, ndhG, ndhI, ndhK, petA, petB, petD, psaB, psaI, psbB, psbC, psbH, rbcL, rpl14, rpoA, rpoC2, rps4, rps11, rps14, rps15, rrn23, tRNA-Arg(UCU), tRNA-Gln(UUG), tRNA-Leu(CAA), tRNA-Pro(UGG), tRNA-Ser(GCU), tRNA-Ser(GGA), ycf2 | atpE, ccsA, cemA, matK, ndhF, ndhH, petN, rpl36, rpoC1, rps3, rps8, tRNA-Ser(UGA), tRNA-Val(UAC), tRNA-Gly(UCC) | clpP, rpl16, rpl20, rpl22, rps16, tRNA-Lys(UUU), ycf3 | ndhA | rpl32 | accD, tRNA-Ala(UGC), ycf1 | tRNA-Leu (UAA) |  |
| **CAN-NUN** | petG, petL, psaJ, psbE, psbF, psbJ, psbL, psbM, psbN, psbT, psbZ, rps12, rps19, rrn4.5, tRNA-Arg(ACG), tRNA-Arg(UCU), tRNA-Asn(GUU), tRNA-Asp(GUC), tRNA-Cys(GCA), tRNA-fM(CAU), tRNA-Glu(UUC), tRNA-His(GUG), tRNA-Ile(CAU), tRNA-Leu(UAG), tRNA-Met(CAU), tRNA-Phe(GAA), tRNA-Ser(GCU), tRNA-Thr(GGU), tRNA-Thr(UGU), tRNA-Trp(CCA), tRNA-Tyr(GUA), tRNA-Val(GAC), tRNA-Gly(GCC), ycf5 (34) | atpI, ndhB, psaA, psaB, psaC, psbA, psbC, psbD, psbI, psbK, rpl2, rpl23, rpl33, rpoB, rps2, rps7, rps11, rps18, rrn5, rrn16, tRNA-Ile(GAU), ycf4 | atpA, atpB, atpF, atpH, cemA, ndhC, ndhD, ndhE, ndhG, ndhH, ndhI, ndhJ, ndhK, petA, petB, petD, petN, psaI, psbB, psbH, rbcL, rpl36, rpoA, rpoC1, rpoC2, rps3, rps4, rps15, rrn23, tRNA-Gln(UUG), tRNA-Leu(CAA), tRNA-Pro(UGG), tRNA-Ser(GGA), ycf2 | atpE, ccsA, matK, ndhF, rpl14, rpl22, rps8, rps14, tRNA-Ser(UGA), tRNA-Val(UAC) | clpP, ndhA, rpl16, rpl20, rps16, tRNA-Lys(UUU), tRNA-Gly(UCC), ycf3 |  | rpl32, ycf1 | accD, tRNA-Ala(UGC) | tRNA-Leu (UAA) |  |
| **CAN-SBU** | petG, petL, petN, psaJ, psbF, psbJ, psbK, psbL, psbN, psbT, psbZ, rps12, rps19, rrn4.5, rrn16, tRNA-Arg(ACG), tRNA-Arg(UCU), tRNA-Asn(GUU), tRNA-Asp(GUC), tRNA-Cys(GCA), tRNA-fM(CAU), tRNA-Glu(UUC), tRNA-His(GUG), tRNA-Ile(CAU), tRNA-Ile(GAU), tRNA-Leu(CAA), tRNA-Leu(UAG), tRNA-Met(CAU), tRNA-Pro(UGG), tRNA-Ser(GCU), tRNA-Ser(GGA), tRNA-Ser(UGA), tRNA-Thr(GGU), tRNA-Thr(UGU), tRNA-Trp(CCA), tRNA-Tyr(GUA), tRNA-Val(GAC), tRNA-Gly(GCC) (38) | atpI, ndhB, ndhC, ndhI, psaA, psaC, psaI, psbA, psbB, psbD, psbE, psbI, psbM, rpl2, rpl23, rps2, rps7, rps18, rrn5, rrn23, tRNA-Ala(UGC) | atpA, atpB, atpF, atpH, ccsA, ndhD, ndhE, ndhF, ndhH, ndhJ, ndhK, petA, psaB, psbC, psbH, rbcL, rpl14, rpoA, rpoB, rpoC1, rpoC2, rps3, rps4, rps8, rps11, rps14, rps15, tRNA-Gln(UUG), tRNA-Phe(GAA), ycf2, ycf4 | atpE, matK, ndhG, petB, petD, rpl16, rpl22, rpl33, tRNA-Lys(UUU), tRNA-Val(UAC), tRNA-Gly(UCC), ycf3 | ndhA, rpl20, rps16 | clpP, rpl36 | ycf1 | accD, cemA, rpl32 | tRNA-Leu (UAA) |  |
| **CAN-SLY** | petG, petN, psaJ, psbF, psbJ, psbK, psbL, psbN, rps12, rps19, rrn4.5, rrn16, tRNA-Arg(ACG), tRNA-Arg(UCU), tRNA-Asn(GUU), tRNA-Asp(GUC), tRNA-Cys(GCA), tRNA-fM(CAU), tRNA-Glu(UUC), tRNA-His(GUG), tRNA-Ile(CAU), tRNA-Ile(GAU), tRNA-Leu(CAA), tRNA-Met(CAU), tRNA-Pro(UGG), tRNA-Ser(GCU), tRNA-Ser(GGA), tRNA-Ser(UGA), tRNA-Thr(GGU), tRNA-Thr(UGU), tRNA-Trp(CCA), tRNA-Tyr(GUA), tRNA-Val(GAC), tRNA-Gly(GCC) (34) | ndhB, ndhC, ndhI, psaA, psaC, psaI, psbA, psbB, psbD, psbE, psbI, psbM, psbZ, rpl2, rpl23, rps2, rps7, rps18, rrn5, rrn23, tRNA-Ala(UGC) | atpA, atpB, atpH, atpI, ndhD, ndhG, ndhH, ndhJ, ndhK, petA, petL, psaB, psbC, rbcL, rpl14, rpl33, rpoA, rpoB, rpoC1, rpoC2, rps4, rps8, rps11, rps14, rps15, tRNA-Gln(UUG), tRNA-Leu(UAG), tRNA-Phe(GAA), ycf2, ycf4 | atpE, atpF, ccsA, ndhE, ndhF, petB, petD, psbH, psbT, rpl22, rps3, tRNA-Val(UAC), ycf3 | matK, ndhA, rpl16, rpl20, rps16, tRNA-Lys(UUU), tRNA-Gly(UCC) | rpl36 | clpP | accD, cemA, rpl32, ycf1 | tRNA-Leu (UAA) |  |
| **CAN-STU** | petG, petL, petN, psaJ, psbF, psbJ, psbK, psbL, psbN, psbT, psbZ, rps12, rps19, rrn4.5, rrn16, tRNA-Arg(ACG), tRNA-Arg(UCU), tRNA-Asn(GUU), tRNA-Asp(GUC), tRNA-Cys(GCA), tRNA-fM(CAU), tRNA-Glu(UUC), tRNA-His(GUG), tRNA-Ile(CAU), tRNA-Ile(GAU), tRNA-Leu(CAA), tRNA-Leu(UAG), tRNA-Met(CAU), tRNA-Pro(UGG), tRNA-Ser(GCU), tRNA-Ser(GGA), tRNA-Ser(UGA), tRNA-Thr(GGU), tRNA-Thr(UGU), tRNA-Trp(CCA), tRNA-Tyr(GUA), tRNA-Val(GAC), tRNA-Gly(GCC) (38) | atpI, ndhB, ndhC, ndhI, psaA, psaC, psaI, psbB, psbD, psbE, psbM, rpl2, rpl23, rps2, rps7, rps18, rrn5, rrn23, tRNA-Ala(UGC) | atpA, atpB, atpF, atpH, ccsA, ndhD, ndhE, ndhF, ndhG, ndhH, ndhJ, ndhK, petA, psaB, psbA, psbC, psbH, psbI, rbcL, rpl14, rpoA, rpoB, rpoC1, rpoC2, rps4, rps8, rps11, rps14, rps15, tRNA-Gln(UUG), tRNA-Phe(GAA), ycf2, ycf4 | atpE, matK, petB, petD, rpl16, rpl22, rpl33, rps3, tRNA-Val(UAC), tRNA-Gly(UCC), ycf3 | ndhA, rpl20, rps16, tRNA-Lys(UUU) | clpP, rpl36 | ycf1 | accD, cemA, rpl32 | tRNA-Leu (UAA) |  |
| **DST-NSY** | petG, petL, psaJ, psbI, psbL, psbM, psbN, psbT, rpl33, rps7, rrn4.5, rrn5, tRNA-Arg(ACG), tRNA-Asn(GUU), tRNA-Cys(GCA), tRNA-fM(CAU), tRNA-Gln(UUG), tRNA-Glu(UUC), tRNA-His(GUG), tRNA-Ile(CAU), tRNA-Leu(UAG), tRNA-Met(CAU), tRNA-Phe(GAA), tRNA-Ser(GCU), tRNA-Thr(GGU), tRNA-Thr(UGU), tRNA-Trp(CCA), tRNA-Tyr(GUA), tRNA-Val(GAC), tRNA-Gly(GCC) (30) | atpH, atpI, ndhB, ndhC, ndhJ, psaA, psaB, psaI, psbA, psbB, psbC, psbD, psbE, psbF, psbJ, psbZ, rbcL, rpl2, rpl23, rpl36, rpoB, rpoC1, rps2, rps4, rps11, rps14, rrn16, rrn23, tRNA-Val(UAC), ycf4 | atpA, atpB, atpE, cemA, ndhD, ndhE, ndhG, ndhH, ndhI, ndhK, petA, petB, petD, petN, psaC, psbH, psbK, rpl14, rpl20, rpoA, rpoC2, rps3, rps8, rps12, rps15, rps18, rps19, tRNA-Arg(UCU), tRNA-Leu(CAA), tRNA-Pro(UGG), tRNA-Ser(GGA), ycf2 | accD, atpF, ccsA, matK, ndhA, ndhF, rpl16, rps16, tRNA-Ile(GAU), tRNA-Lys(UUU), tRNA-Ser(UGA), ycf3 | clpP, rpl22, rpl32, tRNA-Leu(UAA), tRNA-Gly(UCC) | ycf1 | sprA | tRNA-Ala(UGC) |  |  |
| **DST-NTO** | petG, petL, psbI, psbJ, psbL, psbM, psbN, rpl33, rps7, rrn4.5, rrn5, tRNA-Arg(ACG), tRNA-Asn(GUU), tRNA-Cys(GCA), tRNA-fM(CAU), tRNA-Gln(UUG), tRNA-Glu(UUC), tRNA-His(GUG), tRNA-Ile(CAU), tRNA-Leu(UAG), tRNA-Met(CAU), tRNA-Phe(GAA), tRNA-Thr(GGU), tRNA-Thr(UGU), tRNA-Trp(CCA), tRNA-Tyr(GUA), tRNA-Val(GAC), tRNA-Gly(GCC) (28) | atpH, atpI, ndhB, ndhC, ndhG, ndhJ, psaA, psaB, psaI, psaJ, psbA, psbC, psbD, psbE, psbF, psbT, psbZ, rpl2, rpl23, rpoB, rps2, rps4, rps12, rps14, rrn16, rrn23, tRNA-Ile(GAU), ycf4 | atpB, atpE, ndhD, ndhE, ndhH, ndhI, ndhK, petA, psaC, psbB, psbH, psbK, rbcL, rpl14, rpl20, rpl36, rpoA, rpoC2, rps8, rps11, rps18, rps19, tRNA-Leu(CAA), tRNA-Leu(UAA), tRNA-Pro(UGG), tRNA-Ser(GCU), tRNA-Ser(GGA), tRNA-Val(UAC), ycf2 | accD, atpA, atpF, ccsA, cemA, matK, ndhA, ndhF, petB, petD, petN, rpoC1, rps3, rps15, rps16, tRNA-Arg(UCU), tRNA-Ser(UGA), ycf3 | clpP, rpl16, rpl22, rpl32, tRNA-Lys(UUU), tRNA-Gly(UCC) | ycf1 | sprA | tRNA-Ala(UGC) |  |  |
| **DST-NUN** | petG, petL, psaJ, psbI, psbJ, psbL, psbM, psbN, psbT, rpl33, rps7, rps12, rrn4.5, rrn5, tRNA-Arg(ACG), tRNA-Asn(GUU), tRNA-Cys(GCA), tRNA-fM(CAU), tRNA-Gln(UUG), tRNA-Glu(UUC), tRNA-His(GUG), tRNA-Ile(CAU), tRNA-Leu(UAG), tRNA-Met(CAU), tRNA-Phe(GAA), tRNA-Ser(GCU), tRNA-Thr(GGU), tRNA-Thr(UGU), tRNA-Trp(CCA), tRNA-Tyr(GUA), tRNA-Val(GAC), tRNA-Gly(GCC), ycf5 (33) | atpB, atpH, atpI, ndhB, psaA, psaB, psaI, psbA, psbB, psbC, psbD, psbE, psbF, psbZ, rpl2, rpl23, rpl36, rpoB, rps2, rps4, rps11, rrn16, rrn23, tRNA-Ile(GAU), ycf2, ycf4 | atpE, cemA, ndhC, ndhD, ndhE, ndhG, ndhH, ndhI, ndhJ, ndhK, petA, petN, psaC, psbH, psbK, rbcL, rpl14, rpl20, rpoC1, rpoC2, rps3, rps8, rps14, rps18, rps19, tRNA-Arg(UCU), tRNA-Leu(CAA), tRNA-Pro(UGG), tRNA-Ser(GGA), tRNA-Val(UAC) | accD, atpA, atpF, ccsA, matK, ndhA, ndhF, petB, petD, rpoA, rps15, rps16, tRNA-Leu(UAA), tRNA-Lys(UUU), tRNA-Ser(UGA), ycf3 | clpP, rpl16, rpl22, rpl32, tRNA-Gly(UCC) | sprA, ycf1 |  | tRNA-Ala(UGC) |  |  |
| **DST-SBU** | petG, petL, petN, psaI, psaJ, psbI, psbJ, psbL, psbN, psbT, rps7, rps12, rrn4.5, rrn5, rrn16, tRNA-Arg(ACG), tRNA-Asn(GUU), tRNA-Cys(GCA), tRNA-fM(CAU), tRNA-Gln(UUG), tRNA-Glu(UUC), tRNA-His(GUG), tRNA-Ile(CAU), tRNA-Ile(GAU), tRNA-Leu(CAA), tRNA-Leu(UAG), tRNA-Met(CAU), tRNA-Pro(UGG), tRNA-Ser(GCU), tRNA-Ser(GGA), tRNA-Ser(UGA), tRNA-Thr(GGU), tRNA-Thr(UGU), tRNA-Trp(CCA), tRNA-Tyr(GUA), tRNA-Val(GAC), tRNA-Gly(GCC) (37) | atpB, atpH, atpI, ndhB, ndhC, ndhI, psaA, psaB, psbA, psbB, psbD, psbF, psbK, psbM, psbZ, rbcL, rpl2, rpl23, rpoB, rpoC1, rps2, rps14, rrn23, tRNA-Ala(UGC), tRNA-Val(UAC) | atpA, atpE, ndhD, ndhE, ndhG, ndhH, ndhJ, ndhK, petA, psaC, psbC, psbE, psbH, rpl14, rpl20, rpl33, rpoA, rpoC2, rps3, rps4, rps8, rps11, rps18, rps19, tRNA-Arg(UCU), tRNA-Phe(GAA), ycf2, ycf4 | atpF, ccsA, matK, ndhA, ndhF, petB, petD, rps15, tRNA-Leu(UAA), tRNA-Lys(UUU), ycf3 | accD, clpP, rpl16, rpl22, rpl32, rpl36, rps16, sprA, tRNA-Gly(UCC) | ycf1 |  | cemA |  |  |
| **DST-SLY** | petG, petN, psaI, psaJ, psbI, psbJ, psbL, psbN, rps7, rps12, rrn4.5, rrn5, rrn16, tRNA-Arg(ACG), tRNA-Asn(GUU), tRNA-Cys(GCA), tRNA-fM(CAU), tRNA-Gln(UUG), tRNA-Glu(UUC), tRNA-His(GUG), tRNA-Ile(CAU), tRNA-Ile(GAU), tRNA-Leu(CAA), tRNA-Met(CAU), tRNA-Pro(UGG), tRNA-Ser(GCU), tRNA-Ser(GGA), tRNA-Ser(UGA), tRNA-Thr(GGU), tRNA-Thr(UGU), tRNA-Trp(CCA), tRNA-Tyr(GUA), tRNA-Val(GAC), tRNA-Gly(GCC) (34) | atpH, ndhB, ndhC, ndhI, psaA, psaB, psbA, psbB, psbD, psbF, psbK, psbM, rbcL, rpl2, rpl23, rpl33, rps2, rps8, rps14, rrn23, tRNA-Ala(UGC) | atpB, atpE, atpI, ndhD, ndhG, ndhH, ndhJ, ndhK, petA, petL, psaC, psbC, psbE, psbZ, rpl14, rpl20, rpoA, rpoB, rpoC1, rpoC2, rps4, rps11, rps18, rps19, tRNA-Arg(UCU), tRNA-Leu(UAG), tRNA-Phe(GAA), tRNA-Val(UAC), ycf2, ycf4 | atpA, atpF, ccsA, matK, ndhE, ndhF, petB, petD, psbH, psbT, rpl22, rps3, rps15, tRNA-Leu(UAA), ycf3 | ndhA, rpl16, rpl32, rpl36, rps16, tRNA-Lys(UUU), tRNA-Gly(UCC) | accD, clpP, sprA | ycf1 | cemA |  |  |
| **DST-STU** | petG, petL, petN, psaI, psaJ, psbJ, psbL, psbN, psbT, rps7, rps12, rrn4.5, rrn5, rrn16, tRNA-Arg(ACG), tRNA-Asn(GUU), tRNA-Cys(GCA), tRNA-fM(CAU), tRNA-Gln(UUG), tRNA-Glu(UUC), tRNA-His(GUG), tRNA-Ile(CAU), tRNA-Ile(GAU), tRNA-Leu(CAA), tRNA-Leu(UAG), tRNA-Met(CAU), tRNA-Pro(UGG), tRNA-Ser(GCU), tRNA-Ser(GGA), tRNA-Ser(UGA), tRNA-Thr(GGU), tRNA-Thr(UGU), tRNA-Trp(CCA), tRNA-Tyr(GUA), tRNA-Val(GAC), tRNA-Gly(GCC) (36) | atpH, atpI, ndhB, ndhC, ndhI, psaA, psaB, psbA, psbB, psbD, psbF, psbI, psbK, psbM, psbZ, rpl2, rpl23, rpoC1, rps2, rps8, rps14, rrn23, tRNA-Ala(UGC) | atpB, atpE, ccsA, ndhD, ndhE, ndhG, ndhH, ndhJ, ndhK, petA, psaC, psbC, psbE, psbH, rbcL, rpl14, rpl20, rpl33, rpoA, rpoB, rpoC2, rps3, rps4, rps11, rps18, rps19, tRNA-Arg(UCU), tRNA-Phe(GAA), tRNA-Val(UAC), ycf2, ycf4 | atpA, atpF, matK, ndhA, ndhF, petB, petD, rpl16, rps15, tRNA-Leu(UAA), tRNA-Lys(UUU), ycf3 | accD, clpP, rpl22, rpl32, rpl36, rps16, sprA, tRNA-Gly(UCC) | ycf1 |  | cemA |  |  |
| **NSY-NTO** | ndhE, petG, petL, psaI, psbE, psbF, psbH, psbI, psbL, psbM, psbN, psbZ, rpl20, rpl23, rpl33, rps7, rps18, rrn4.5, rrn5, rrn16, tRNA-Ala(UGC), tRNA-Arg(ACG), tRNA-Asn(GUU), tRNA-Asp(GUC), tRNA-Cys(GCA), tRNA-fM(CAU), tRNA-Gln(UUG), tRNA-Glu(UUC), tRNA-His(GUG), tRNA-Ile(CAU), tRNA-Leu(CAA), tRNA-Leu(UAG), tRNA-Met(CAU), tRNA-Phe(GAA), tRNA-Pro(UGG), tRNA-Ser(GGA), tRNA-Ser(UGA), tRNA-Thr(GGU), tRNA-Thr(UGU), tRNA-Trp(CCA), tRNA-Tyr(GUA), tRNA-Val(GAC), tRNA-Gly(GCC) (43) | accD, atpA, atpE, atpH, atpI, ccsA, ndhB, ndhC, ndhD, ndhG, ndhH, ndhI, ndhJ, ndhK, petA, petB, petD, psaA, psaB, psaC, psaJ, psbA, psbB, psbC, psbD, psbJ, psbK, psbT, rbcL, rpl2, rpl14, rpl36, rpoB, rpoC2, rps2, rps4, rps8, rps11, rps14, rps19, rrn23, sprA, tRNA-Val(UAC), ycf2, ycf4 | atpB, atpF, cemA, clpP, matK, ndhA, ndhF, petN, rpl16, rpl22, rpl32, rpoA, rpoC1, rps3, rps15, rps16, tRNA-Arg(UCU), tRNA-Ile(GAU), tRNA-Leu(UAA), tRNA-Lys(UUU), tRNA-Ser(GCU), tRNA-Gly(UCC), ycf3 | rps12, ycf1 |  |  |  |  |  |  |
| **NSY-NUN** | atpI, petG, petL, petN, psaC, psaI, psaJ, psbE, psbF, psbH, psbI, psbL, psbM, psbN, psbT, psbZ, rpl20, rpl23, rpl33, rpl36, rps7, rps11, rps18, rrn4.5, rrn5, rrn16, rrn23, tRNA-Ala(UGC), tRNA-Arg(ACG), tRNA-Arg(UCU), tRNA-Asn(GUU), tRNA-Asp(GUC), tRNA-Cys(GCA), tRNA-fM(CAU), tRNA-Gln(UUG), tRNA-Glu(UUC), tRNA-His(GUG), tRNA-Ile(CAU), tRNA-Leu(CAA), tRNA-Leu(UAG), tRNA-Met(CAU), tRNA-Phe(GAA), tRNA-Pro(UGG), tRNA-Ser(GCU), tRNA-Ser(GGA), tRNA-Ser(UGA), tRNA-Thr(GGU), tRNA-Thr(UGU), tRNA-Trp(CCA), tRNA-Tyr(GUA), tRNA-Val(GAC), tRNA-Gly(GCC) (52) | accD, atpA, atpB, atpE, atpF, atpH, ccsA, cemA, matK, ndhA, ndhB, ndhD, ndhE, ndhF, ndhG, ndhH, ndhI, ndhJ, ndhK, petA, petB, petD, psaA, psaB, psbA, psbB, psbC, psbD, psbJ, psbK, rbcL, rpl2, rpl14, rpoA, rpoB, rpoC1, rpoC2, rps2, rps3, rps4, rps8, rps14, rps16, rps19, sprA, tRNA-Val(UAC), ycf2, ycf4 | clpP, ndhC, rpl16, rpl22, rpl32, rps12, rps15, tRNA-Ile(GAU), tRNA-Lys(UUU), tRNA-Gly(UCC), ycf1, ycf3 | tRNA-Leu(UAA) |  |  |  |  |  |  |
| **NSY-SBU** | petG, petL, psaJ, psbF, psbI, psbL, psbN, psbT, psbZ, rpl23, rps7, rrn4.5, rrn5, tRNA-Arg(ACG), tRNA-Arg(UCU), tRNA-Asn(GUU), tRNA-Asp(GUC), tRNA-Cys(GCA), tRNA-fM(CAU), tRNA-Gln(UUG), tRNA-Glu(UUC), tRNA-His(GUG), tRNA-Ile(CAU), tRNA-Leu(UAG), tRNA-Met(CAU), tRNA-Ser(GCU), tRNA-Thr(GGU), tRNA-Thr(UGU), tRNA-Trp(CCA), tRNA-Tyr(GUA), tRNA-Val(GAC), tRNA-Gly(GCC) (32) | atpI, ndhB, ndhC, ndhI, psaA, psaC, psaI, psbA, psbB, psbD, psbE, psbJ, psbM, rpl2, rpl20, rpoB, rpoC1, rps2, rps14, rps18, rps19, rrn16, rrn23, ycf2 | atpA, atpB, atpE, atpF, atpH, ndhD, ndhE, ndhG, ndhH, ndhJ, ndhK, petA, petB, petD, petN, psaB, psbC, psbH, psbK, rbcL, rpl14, rpl33, rpoC2, rps3, rps4, rps8, rps11, rps12, rps15, tRNA-Leu(CAA), tRNA-Phe(GAA), tRNA-Pro(UGG), tRNA-Ser(GGA), tRNA-Val(UAC), tRNA-Gly(UCC), ycf4 | ccsA, matK, ndhA, ndhF, rpl16, rpl22, rpl32, rpoA, tRNA-Ile(GAU), tRNA-Lys(UUU), tRNA-Ser(UGA), ycf3 | accD, rpl36, rps16, tRNA-Leu(UAA) | clpP, ycf1 | sprA | cemA, tRNA-Ala(UGC) |  |  |
| **NSY-SLY** | petG, psaJ, psbF, psbI, psbL, psbN, rpl23, rps7, rrn4.5, rrn5, tRNA-Arg(ACG), tRNA-Arg(UCU), tRNA-Asn(GUU), tRNA-Asp(GUC), tRNA-Cys(GCA), tRNA-fM(CAU), tRNA-Gln(UUG), tRNA-Glu(UUC), tRNA-His(GUG), tRNA-Ile(CAU), tRNA-Met(CAU), tRNA-Ser(GCU), tRNA-Thr(GGU), tRNA-Thr(UGU), tRNA-Trp(CCA), tRNA-Tyr(GUA), tRNA-Val(GAC), tRNA-Gly(GCC) (28) | ndhB, ndhC, ndhI, psaA, psaC, psaI, psbD, psbE, psbJ, psbM, psbZ, rpl2, rpl20, rpl33, rps2, rps14, rps18, rps19, rrn16, rrn23, ycf2 | atpA, atpB, atpE, atpH, atpI, ndhD, ndhE, ndhG, ndhH, ndhJ, ndhK, petA, petB, petL, petN, psaB, psbA, psbB, psbC, psbK, rbcL, rpl14, rpoA, rpoB, rpoC1, rps4, rps8, rps11, rps12, rps15, tRNA-Leu(CAA), tRNA-Leu(UAG), tRNA-Phe(GAA), tRNA-Pro(UGG), tRNA-Ser(GGA), tRNA-Val(UAC), ycf4 | atpF, matK, ndhF, petD, psbH, psbT, rpl22, rpoC2, rps3, tRNA-Ile(GAU), tRNA-Ser(UGA), tRNA-Gly(UCC) | accD, ccsA, ndhA, rpl16, rpl32, rpl36, rps16, tRNA-Leu(UAA), tRNA-Lys(UUU), ycf3 | ycf1 | clpP | cemA, sprA, tRNA-Ala(UGC) |  |  |
| **NSY-STU** | petG, petL, psaJ, psbF, psbL, psbN, psbT, psbZ, rpl23, rps7, rrn4.5, rrn5, tRNA-Arg(ACG), tRNA-Arg(UCU), tRNA-Asn(GUU), tRNA-Asp(GUC), tRNA-Cys(GCA), tRNA-fM(CAU), tRNA-Gln(UUG), tRNA-Glu(UUC), tRNA-His(GUG), tRNA-Ile(CAU), tRNA-Leu(UAG), tRNA-Met(CAU), tRNA-Ser(GCU), tRNA-Thr(GGU), tRNA-Thr(UGU), tRNA-Trp(CCA), tRNA-Tyr(GUA), tRNA-Val(GAC), tRNA-Gly(GCC) (31) | atpI, ndhB, ndhC, ndhE, ndhI, psaA, psaC, psaI, psbA, psbB, psbD, psbE, psbI, psbJ, psbM, rpl2, rpl20, rpoB, rps2, rps18, rps19, rrn16, rrn23, ycf2 | atpA, atpB, atpE, atpF, atpH, ndhD, ndhG, ndhH, ndhJ, ndhK, petA, petB, petN, psaB, psbC, psbH, psbK, rbcL, rpl14, rpl33, rpoA, rpoC1, rpoC2, rps3, rps4, rps8, rps11, rps12, rps14, rps15, tRNA-Leu(CAA), tRNA-Phe(GAA), tRNA-Pro(UGG), tRNA-Ser(GGA), tRNA-Val(UAC), tRNA-Gly(UCC), ycf4 | ccsA, matK, ndhA, ndhF, petD, rpl16, rpl22, rpl32, tRNA-Ile(GAU), tRNA-Leu(UAA), tRNA-Lys(UUU), tRNA-Ser(UGA) | accD, rpl36, rps16, ycf3 | clpP, ycf1 | sprA | cemA, tRNA-Ala(UGC) |  |  |
| **NTO-NUN** | petG, petL, psaI, psbE, psbF, psbH, psbI, psbJ, psbK, psbL, psbM, psbN, psbZ, rpl20, rpl23, rpl33, rps7, rps18, rrn4.5, rrn5, rrn16, tRNA-Ala(UGC), tRNA-Arg(ACG), tRNA-Asn(GUU), tRNA-Asp(GUC), tRNA-Cys(GCA), tRNA-fM(CAU), tRNA-Gln(UUG), tRNA-Glu(UUC), tRNA-His(GUG), tRNA-Ile(CAU), tRNA-Ile(GAU), tRNA-Leu(CAA), tRNA-Leu(UAG), tRNA-Met(CAU), tRNA-Phe(GAA), tRNA-Pro(UGG), tRNA-Ser(GGA), tRNA-Ser(UGA), tRNA-Thr(GGU), tRNA-Thr(UGU), tRNA-Trp(CCA), tRNA-Tyr(GUA), tRNA-Val(GAC), tRNA-Gly(GCC) (45) | atpA, atpB, atpE, atpH, atpI, clpP, ndhB, ndhD, ndhE, ndhG, ndhH, ndhI, ndhJ, ndhK, petB, psaA, psaB, psaC, psaJ, psbA, psbB, psbC, psbD, psbT, rbcL, rpl2, rpl36, rpoB, rpoC2, rps2, rps4, rps8, rps11, rps12, rps19, rrn23, sprA, tRNA-Leu(UAA), tRNA-Val(UAC), ycf2, ycf4 | accD, atpF, ccsA, cemA, matK, ndhA, ndhC, ndhF, petA, petD, petN, rpl14, rpl16, rpl22, rpl32, rpoA, rpoC1, rps3, rps14, rps15, rps16, tRNA-Arg(UCU), tRNA-Lys(UUU), tRNA-Ser(GCU), tRNA-Gly(UCC), ycf3 | ycf1 |  |  |  |  |  |  |
| **NTO-SBU** | petG, petL, psbF, psbI, psbJ, psbL, psbN, psbZ, rpl23, rps7, rrn4.5, rrn5, tRNA-Arg(ACG), tRNA-Asn(GUU), tRNA-Asp(GUC), tRNA-Cys(GCA), tRNA-fM(CAU), tRNA-Gln(UUG), tRNA-Glu(UUC), tRNA-His(GUG), tRNA-Ile(CAU), tRNA-Leu(UAG), tRNA-Met(CAU), tRNA-Thr(GGU), tRNA-Thr(UGU), tRNA-Trp(CCA), tRNA-Tyr(GUA), tRNA-Val(GAC), tRNA-Gly(GCC) (29) | ndhB, ndhC, psaA, psaC, psaI, psaJ, psbB, psbD, psbE, psbK, psbM, psbT, rpl2, rpl20, rps2, rps12, rps14, rps18, rps19, rrn16, rrn23, tRNA-Ile(GAU), ycf2 | atpA, atpB, atpE, atpH, atpI, ndhD, ndhE, ndhG, ndhH, ndhI, ndhJ, ndhK, petB, psaB, psbA, psbC, psbH, rbcL, rpl14, rpl33, rpoB, rpoC2, rps4, rps8, tRNA-Arg(UCU), tRNA-Leu(CAA), tRNA-Leu(UAA), tRNA-Phe(GAA), tRNA-Pro(UGG), tRNA-Ser(GCU), tRNA-Ser(GGA), tRNA-Val(UAC), ycf4 | atpF, ccsA, matK, ndhF, petA, petD, petN, rpl22, rpoA, rpoC1, rps3, rps11, rps15, tRNA-Ser(UGA), tRNA-Gly(UCC) | accD, clpP, ndhA, rpl16, rpl32, rpl36, rps16, tRNA-Lys(UUU), ycf3 | ycf1 | sprA | cemA, tRNA-Ala(UGC) |  |  |
| **NTO-SLY** | petG, psbF, psbI, psbJ, psbL, psbN, rpl23, rps7, rrn4.5, rrn5, tRNA-Arg(ACG), tRNA-Asn(GUU), tRNA-Asp(GUC), tRNA-Cys(GCA), tRNA-fM(CAU), tRNA-Gln(UUG), tRNA-Glu(UUC), tRNA-His(GUG), tRNA-Ile(CAU), tRNA-Met(CAU), tRNA-Thr(GGU), tRNA-Thr(UGU), tRNA-Trp(CCA), tRNA-Tyr(GUA), tRNA-Val(GAC), tRNA-Gly(GCC) (26) | ndhB, ndhC, psaC, psaI, psaJ, psbD, psbE, psbK, psbM, psbZ, rpl2, rpl20, rpl33, rps2, rps12, rps14, rps18, rps19, rrn16, rrn23, tRNA-Ile(GAU), ycf2 | atpA, atpB, atpE, atpH, atpI, ndhD, ndhE, ndhG, ndhI, ndhJ, ndhK, petB, petL, psaA, psaB, psbA, psbB, psbC, rbcL, rpl14, rpoB, rps4, rps8, tRNA-Arg(UCU), tRNA-Leu(CAA), tRNA-Leu(UAA), tRNA-Leu(UAG), tRNA-Phe(GAA), tRNA-Pro(UGG), tRNA-Ser(GCU), tRNA-Ser(GGA), tRNA-Val(UAC), ycf4 | atpF, ndhF, ndhH, petA, petD, petN, psbH, rpl22, rpoA, rpoC1, rpoC2, rps11, rps15, tRNA-Ser(UGA), tRNA-Gly(UCC) | accD, ccsA, matK, ndhA, psbT, rpl16, rpl32, rpl36, rps3, rps16, tRNA-Lys(UUU), ycf3 | clpP, ycf1 |  | cemA, sprA, tRNA-Ala(UGC) |  |  |
| **NTO-STU** | petG, petL, psbF, psbJ, psbL, psbN, psbZ, rpl23, rps7, rrn4.5, rrn5, tRNA-Arg(ACG), tRNA-Asn(GUU), tRNA-Asp(GUC), tRNA-Cys(GCA), tRNA-fM(CAU), tRNA-Gln(UUG), tRNA-Glu(UUC), tRNA-His(GUG), tRNA-Ile(CAU), tRNA-Leu(UAG), tRNA-Met(CAU), tRNA-Thr(GGU), tRNA-Thr(UGU), tRNA-Trp(CCA), tRNA-Tyr(GUA), tRNA-Val(GAC), tRNA-Gly(GCC) (28) | atpI, ndhB, ndhC, ndhE, psaA, psaC, psaI, psaJ, psbB, psbD, psbE, psbI, psbK, psbM, psbT, rpl2, rpl20, rps2, rps12, rps18, rps19, rrn16, rrn23, tRNA-Ile(GAU), ycf2 | atpA, atpB, atpE, atpH, ndhD, ndhG, ndhI, ndhJ, ndhK, petB, psaB, psbA, psbC, psbH, rbcL, rpl14, rpl33, rpoB, rpoC2, rps4, rps8, rps14, tRNA-Arg(UCU), tRNA-Leu(CAA), tRNA-Leu(UAA), tRNA-Phe(GAA), tRNA-Pro(UGG), tRNA-Ser(GCU), tRNA-Ser(GGA), tRNA-Val(UAC), ycf4 | atpF, ccsA, matK, ndhF, ndhH, petA, petD, petN, rpl16, rpl32, rpoA, rpoC1, rps3, rps11, rps15, tRNA-Ser(UGA), tRNA-Gly(UCC) | accD, clpP, ndhA, rpl22, rpl36, rps16, tRNA-Lys(UUU), ycf3 | ycf1 | sprA | cemA, tRNA-Ala(UGC) |  |  |
| **NUN-SBU** | petG, petL, psaJ, psbF, psbI, psbJ, psbL, psbN, psbT, psbZ, rpl23, rps7, rps12, rps19, rrn4.5, rrn5, tRNA-Arg(ACG), tRNA-Arg(UCU), tRNA-Asn(GUU), tRNA-Asp(GUC), tRNA-Cys(GCA), tRNA-fM(CAU), tRNA-Gln(UUG), tRNA-Glu(UUC), tRNA-His(GUG), tRNA-Ile(CAU), tRNA-Leu(UAG), tRNA-Met(CAU), tRNA-Ser(GCU), tRNA-Thr(GGU), tRNA-Thr(UGU), tRNA-Trp(CCA), tRNA-Tyr(GUA), tRNA-Val(GAC), tRNA-Gly(GCC) (35) | atpB, atpH, atpI, ndhB, ndhI, psaA, psaC, psaI, psbA, psbB, psbD, psbE, psbK, psbM, rpl2, rpl20, rpoB, rps2, rps18, rrn16, rrn23, tRNA-Ile(GAU), ycf2, ycf4 | atpA, atpE, atpF, ndhC, ndhD, ndhE, ndhH, ndhJ, ndhK, petA, petB, petN, psaB, psbC, psbH, rbcL, rpl14, rpl33, rpoC1, rpoC2, rps3, rps4, rps8, rps11, rps14, tRNA-Leu(CAA), tRNA-Leu(UAA), tRNA-Phe(GAA), tRNA-Pro(UGG), tRNA-Ser(GGA), tRNA-Val(UAC) | ccsA, matK, ndhA, ndhF, ndhG, petD, rpl16, rpl22, rpl32, rpoA, rps15, tRNA-Ser(UGA), tRNA-Gly(UCC) | accD, clpP, rpl36, rps16, tRNA-Lys(UUU), ycf3 | ycf1 | sprA | cemA, tRNA-Ala(UGC) |  |  |
| **NUN-SLY** | petG, psaJ, psbF, psbI, psbJ, psbL, psbN, rpl23, rps7, rps12, rps19, rrn4.5, rrn5, tRNA-Arg(ACG), tRNA-Arg(UCU), tRNA-Asn(GUU), tRNA-Asp(GUC), tRNA-Cys(GCA), tRNA-fM(CAU), tRNA-Gln(UUG), tRNA-Glu(UUC), tRNA-His(GUG), tRNA-Ile(CAU), tRNA-Met(CAU), tRNA-Ser(GCU), tRNA-Thr(GGU), tRNA-Thr(UGU), tRNA-Trp(CCA), tRNA-Tyr(GUA), tRNA-Val(GAC), tRNA-Gly(GCC) (31) | atpH, ndhB, ndhI, psaA, psaC, psaI, psbD, psbE, psbK, psbM, psbZ, rpl2, rpl20, rpl33, rps2, rps18, rrn16, rrn23, tRNA-Ile(GAU), ycf2 | atpA, atpB, atpE, atpI, ndhC, ndhD, ndhH, ndhJ, ndhK, petA, petB, petL, petN, psaB, psbA, psbB, psbC, rbcL, rpl14, rpoB, rpoC1, rps4, rps8, rps11, rps14, tRNA-Leu(CAA), tRNA-Leu(UAG), tRNA-Phe(GAA), tRNA-Pro(UGG), tRNA-Ser(GGA), tRNA-Val(UAC), ycf4 | atpF, matK, ndhE, ndhF, ndhG, petD, psbH, psbT, rpl22, rpoA, rpoC2, rps3, rps15, tRNA-Leu(UAA), tRNA-Ser(UGA), tRNA-Gly(UCC) | accD, ccsA, ndhA, rpl16, rpl32, rpl36, rps16, tRNA-Lys(UUU), ycf3 | clpP, ycf1 |  | cemA, sprA, tRNA-Ala(UGC) |  |  |
| **NUN-STU** | petG, petL, psaJ, psbF, psbJ, psbL, psbN, psbT, psbZ, rpl23, rps7, rps12, rps19, rrn4.5, rrn5, tRNA-Arg(ACG), tRNA-Arg(UCU), tRNA-Asn(GUU), tRNA-Asp(GUC), tRNA-Cys(GCA), tRNA-fM(CAU), tRNA-Gln(UUG), tRNA-Glu(UUC), tRNA-His(GUG), tRNA-Ile(CAU), tRNA-Leu(UAG), tRNA-Met(CAU), tRNA-Ser(GCU), tRNA-Thr(GGU), tRNA-Thr(UGU), tRNA-Trp(CCA), tRNA-Tyr(GUA), tRNA-Val(GAC), tRNA-Gly(GCC) (34) | atpI, ndhB, ndhI, psaA, psaC, psaI, psbB, psbD, psbE, psbI, psbK, psbM, rpl2, rpl20, rps2, rps18, rrn16, rrn23, tRNA-Ile(GAU), ycf2, ycf4 | atpA, atpB, atpE, atpF, atpH, ndhC, ndhD, ndhE, ndhH, ndhJ, ndhK, petA, petB, petN, psaB, psbA, psbC, psbH, rbcL, rpl14, rpl33, rpoB, rpoC1, rpoC2, rps3, rps4, rps8, rps11, rps14, tRNA-Leu(CAA), tRNA-Leu(UAA), tRNA-Phe(GAA), tRNA-Pro(UGG), tRNA-Ser(GGA), tRNA-Val(UAC) | ccsA, matK, ndhF, ndhG, petD, rpl16, rpl22, rpl32, rpoA, rps15, tRNA-Lys(UUU), tRNA-Ser(UGA), tRNA-Gly(UCC) | accD, clpP, ndhA, rpl36, rps16, ycf3 | ycf1 | sprA | cemA, tRNA-Ala(UGC) |  |  |
| **SBU-SLY** | atpE, petG, petN, psaC, psaI, psaJ, psbE, psbF, psbI, psbJ, psbK, psbL, psbM, psbN, rpl2, rpl20, rpl23, rpl36, rps4, rps7, rps12, rps14, rps18, rps19, rrn4.5, rrn5, rrn16, rrn23, tRNA-Ala(UGC), tRNA-Arg(ACG), tRNA-Arg(UCU), tRNA-Asn(GUU), tRNA-Asp(GUC), tRNA-Cys(GCA), tRNA-fM(CAU), tRNA-Gln(UUG), tRNA-Glu(UUC), tRNA-His(GUG), tRNA-Ile(CAU), tRNA-Ile(GAU), tRNA-Leu(CAA), tRNA-Met(CAU), tRNA-Phe(GAA), tRNA-Pro(UGG), tRNA-Ser(GCU), tRNA-Ser(GGA), tRNA-Ser(UGA), tRNA-Thr(GGU), tRNA-Thr(UGU), tRNA-Trp(CCA), tRNA-Tyr(GUA), tRNA-Val(GAC), tRNA-Gly(GCC) (53) | accD, atpA, atpB, atpH, atpI, cemA, ndhB, ndhC, ndhD, ndhF, ndhG, ndhH, ndhI, ndhJ, ndhK, petA, petB, petD, psaA, psaB, psbA, psbB, psbC, psbD, psbH, psbZ, rbcL, rpl14, rpl22, rpoA, rpoB, rpoC1, rpoC2, rps2, rps3, rps8, rps11, tRNA-Leu(UAA), tRNA-Val(UAC), ycf2, ycf3, ycf4 | atpF, ccsA, matK, ndhE, petL, rpl16, rpl32, rpl33, rps15, rps16, tRNA-Leu(UAG), tRNA-Lys(UUU), tRNA-Gly(UCC) | ndhA, psbT, ycf1 | clpP, sprA |  |  |  |  |  |
| **SBU-STU** | atpE, cemA, ndhI, petG, petL, petN, psaC, psaI, psaJ, psbE, psbF, psbH, psbJ, psbK, psbL, psbM, psbN, psbT, psbZ, rpl2, rpl20, rpl23, rpl33, rpl36, rps2, rps4, rps7, rps12, rps18, rps19, rrn4.5, rrn5, rrn16, rrn23, sprA, tRNA-Ala(UGC), tRNA-Arg(ACG), tRNA-Arg(UCU), tRNA-Asn(GUU), tRNA-Asp(GUC), tRNA-Cys(GCA), tRNA-fM(CAU), tRNA-Gln(UUG), tRNA-Glu(UUC), tRNA-His(GUG), tRNA-Ile(CAU), tRNA-Ile(GAU), tRNA-Leu(CAA), tRNA-Leu(UAG), tRNA-Met(CAU), tRNA-Phe(GAA), tRNA-Pro(UGG), tRNA-Ser(GCU), tRNA-Ser(GGA), tRNA-Ser(UGA), tRNA-Thr(GGU), tRNA-Thr(UGU), tRNA-Trp(CCA), tRNA-Tyr(GUA), tRNA-Val(GAC), tRNA-Gly(GCC), ycf4 (62) | accD, atpA, atpB, atpF, atpI, ccsA, clpP, matK, ndhA, ndhB, ndhC, ndhD, ndhE, ndhF, ndhG, ndhH, ndhJ, ndhK, petA, petB, petD, psaA, psaB, psbA, psbB, psbC, psbD, psbI, rbcL, rpl14, rpl16, rpl22, rpl32, rpoA, rpoB, rpoC1, rpoC2, rps3, rps8, rps11, rps14, rps15, rps16, tRNA-Leu(UAA), tRNA-Lys(UUU), tRNA-Val(UAC), tRNA-Gly(UCC), ycf1, ycf2, ycf3 | atpH |  |  |  |  |  |  |  |
| **SLY-STU** | atpE, petG, petN, psaC, psaI, psaJ, psbE, psbF, psbJ, psbK, psbL, psbM, psbN, rpl2, rpl20, rpl23, rpl36, rps4, rps7, rps11, rps12, rps18, rps19, rrn4.5, rrn5, rrn16, rrn23, tRNA-Ala(UGC), tRNA-Arg(ACG), tRNA-Arg(UCU), tRNA-Asn(GUU), tRNA-Asp(GUC), tRNA-Cys(GCA), tRNA-fM(CAU), tRNA-Gln(UUG), tRNA-Glu(UUC), tRNA-His(GUG), tRNA-Ile(CAU), tRNA-Ile(GAU), tRNA-Leu(CAA), tRNA-Met(CAU), tRNA-Phe(GAA), tRNA-Pro(UGG), tRNA-Ser(GCU), tRNA-Ser(GGA), tRNA-Ser(UGA), tRNA-Thr(GGU), tRNA-Thr(UGU), tRNA-Trp(CCA), tRNA-Tyr(GUA), tRNA-Val(GAC), tRNA-Gly(GCC) (52) | accD, atpA, atpB, atpF, atpI, ccsA, cemA, ndhB, ndhC, ndhD, ndhE, ndhF, ndhG, ndhH, ndhI, ndhJ, ndhK, petA, petB, petD, psaA, psaB, psbA, psbB, psbC, psbD, psbH, psbI, psbZ, rpl14, rpl22, rpoA, rpoB, rpoC1, rpoC2, rps2, rps3, rps8, rps14, tRNA-Leu(UAA), ycf2, ycf3, ycf4 | atpH, matK, petL, rbcL, rpl16, rpl32, rpl33, rps15, rps16, tRNA-Leu(UAG), tRNA-Lys(UUU), tRNA-Val(UAC), tRNA-Gly(UCC) | ndhA, psbT, ycf1 | clpP, sprA |  |  |  |  |  |

ABE- *Atropa belladonna*, CAN- *Capsicum annuum*, DST- *Datura stramonium*, NSY- *Nicotiana sylvestris*, NTA- *Nicotiana tabacum*, NTO- *Nicotiana tomentosiformis*, NUN- *Nicotiana undulata*, SBU- *Solanum bulbocastanum*, SLY- *Solanum lycopersicum*, STU- *Solanum tuberosum*.
